# Artificial intelligence-assisted quantification and assessment of whole slide images for pediatric kidney disease diagnosis

**DOI:** 10.1093/bioinformatics/btad740

**Published:** 2023-12-07

**Authors:** Chunyue Feng, Kokhaur Ong, David M Young, Bingxian Chen, Longjie Li, Xinmi Huo, Haoda Lu, Weizhong Gu, Fei Liu, Hongfeng Tang, Manli Zhao, Min Yang, Kun Zhu, Limin Huang, Qiang Wang, Gabriel Pik Liang Marini, Kun Gui, Hao Han, Stephan J Sanders, Lin Li, Weimiao Yu, Jianhua Mao

**Affiliations:** Department of Nephrology, Children’s Hospital, Zhejiang University School of Medicine, Hangzhou 310000, China; National Clinical Research Center for Child Health, Hangzhou 310000, China; Bioinformatics Institute, A*STAR, Singapore 138673, Singapore; Institute of Molecular and Cell Biology, A*STAR, Singapore 138673, Singapore; Department of Psychiatry and Behavioral Sciences, UCSF Weill Institute for Neurosciences, University of California, San Francisco, CA, 94143, United States; Ningbo Konfoong Bioinformation Tech Co., Ltd., Ningbo 315000, China; Bioinformatics Institute, A*STAR, Singapore 138673, Singapore; Bioinformatics Institute, A*STAR, Singapore 138673, Singapore; Bioinformatics Institute, A*STAR, Singapore 138673, Singapore; Institute for AI in Medicine, Nanjing University of Information Science and Technology, Nanjing 210044, China; National Clinical Research Center for Child Health, Hangzhou 310000, China; Department of Pathology, Children’s Hospital, Zhejiang University School of Medicine, Hangzhou, 310000, China; Department of Nephrology, Children’s Hospital, Zhejiang University School of Medicine, Hangzhou 310000, China; National Clinical Research Center for Child Health, Hangzhou 310000, China; National Clinical Research Center for Child Health, Hangzhou 310000, China; Department of Pathology, Children’s Hospital, Zhejiang University School of Medicine, Hangzhou, 310000, China; National Clinical Research Center for Child Health, Hangzhou 310000, China; Department of Pathology, Children’s Hospital, Zhejiang University School of Medicine, Hangzhou, 310000, China; National Clinical Research Center for Child Health, Hangzhou 310000, China; Department of Pathology, Children’s Hospital, Zhejiang University School of Medicine, Hangzhou, 310000, China; National Clinical Research Center for Child Health, Hangzhou 310000, China; Department of Pathology, Children’s Hospital, Zhejiang University School of Medicine, Hangzhou, 310000, China; Department of Nephrology, Children’s Hospital, Zhejiang University School of Medicine, Hangzhou 310000, China; National Clinical Research Center for Child Health, Hangzhou 310000, China; Ningbo Konfoong Bioinformation Tech Co., Ltd., Ningbo 315000, China; Bioinformatics Institute, A*STAR, Singapore 138673, Singapore; Ningbo Konfoong Bioinformation Tech Co., Ltd., Ningbo 315000, China; Institute of Molecular and Cell Biology, A*STAR, Singapore 138673, Singapore; Department of Psychiatry and Behavioral Sciences, UCSF Weill Institute for Neurosciences, University of California, San Francisco, CA, 94143, United States; Department of Nephrology, Shanghai Changzheng Hospital, Naval Medical University, Shanghai 200003, China; Bioinformatics Institute, A*STAR, Singapore 138673, Singapore; Institute of Molecular and Cell Biology, A*STAR, Singapore 138673, Singapore; Institute for AI in Medicine, Nanjing University of Information Science and Technology, Nanjing 210044, China; Department of Nephrology, Children’s Hospital, Zhejiang University School of Medicine, Hangzhou 310000, China; National Clinical Research Center for Child Health, Hangzhou 310000, China

## Abstract

**Motivation:**

Pediatric kidney disease is a widespread, progressive condition that severely impacts growth and development of children. Chronic kidney disease is often more insidious in children than in adults, usually requiring a renal biopsy for diagnosis. Biopsy evaluation requires copious examination by trained pathologists, which can be tedious and prone to human error. In this study, we propose an artificial intelligence (AI) method to assist pathologists in accurate segmentation and classification of pediatric kidney structures, named as AI-based Pediatric Kidney Diagnosis (APKD).

**Results:**

We collected 2935 pediatric patients diagnosed with kidney disease for the development of APKD. The dataset comprised 93 932 histological structures annotated manually by three skilled nephropathologists. APKD scored an average accuracy of 94% for each kidney structure category, including 99% in the glomerulus. We found strong correlation between the model and manual detection in detected glomeruli (Spearman correlation coefficient *r* = 0.98, *P* < .001; intraclass correlation coefficient ICC = 0.98, 95% CI = 0.96–0.98). Compared to manual detection, APKD was approximately 5.5 times faster in segmenting glomeruli. Finally, we show how the pathological features extracted by APKD can identify focal abnormalities of the glomerular capillary wall to aid in the early diagnosis of pediatric kidney disease.

**Availability and implementation:**

https://github.com/ChunyueFeng/Kidney-DataSet.

## 1 Introduction

Chronic kidney disease (CKD) is a global healthcare challenge, with a prevalence of 13.4% worldwide and 10.8% in China (Zhang *et al.* 2012, Lv and Zhang 2019). This affects around 120 million people and places a substantial burden on both patients and society. Pediatric CKD, characterized by symptoms like hematuria, proteinuria, anemia, and growth retardation, saw rising rates of end-stage renal disease. While early diagnosis and treatments stabilized this trend, recent data show a concerning increase in pediatric CKD inpatients in China from 1.93% in 2013 to 2.09% in 2016, necessitating long-term monitoring and intervention (Becherucci *et al.* 2016, Kaspar *et al.* 2016, Shi *et al.* 2021).

Pediatric CKD is often elusive, requiring renal biopsies for precise evaluation and diagnosis. These biopsies involve sampling kidney tissue to understand disease specifics, guide treatments, and predict disease progresses. However, their low specificity means detailed pathological interpretation by expert nephropathologists is essential, demanding significant time and resources. With the advent of whole slide imaging (WSI) techniques, physical slides are digitized, but analyzing multiple slides for each patient remains a labor-intensive task. Moreover, we are facing a shortage of pathologists globally.

Advancements in computer science and imaging technology have enabled the practical use of artificial intelligence (AI) in various medical expert systems, including clinical pathology diagnosis using WSI analysis. In the field of clinical renal pathological diagnosis, the application value of AI is mainly reflected in the following aspects. (i) AI interprets pathological slides, extracting vital quantitative metrics, enhancing diagnostic efficiency. (ii) Deep learning (DL) on vast digital pathology and clinical data enhances disease diagnosis. (iii) AI enables standardized quantification, bridging clinical and pathological interpretations. AI excels in measuring renal pathology features across WSIs, offering enhanced reliability of assessments such as glomerular and tubular and reducing diagnostic errors and variations.

In recent years, there has been a surge in kidney pathology studies using DL. Initially, these studies focused on determining glomerular assessment (Kakimoto *et al.* 2014, Kato *et al.* 2015, Marée *et al.* 2016, Sarder *et al.* 2016, Gadermayr *et al.* 2017, 2019a, 2019b, Temerinac-Ott *et al.* 2017, Gallego *et al.* 2018, 2021, Marsh *et al.* 2018, 2021, Simon *et al.* 2018, Ginley *et al.* 2019), including distinguishing sclerotic and non-sclerotic glomeruli (Bukowy *et al.* 2018, Zee *et al.* 2018, Kannan *et al.* 2019, Bueno *et al.* 2020a,b). Refinements include precise segmentation with high precision, recall, and F-measure scores (Barros *et al.* 2017, Sheehan and Korstanje 2018, Sheehan *et al.* 2019, Chagas *et al.* 2020). DL-based segmentation expanded to various kidney structures like tubules, capillaries, and arterioles, covering pathologies such as interstitial fibrosis and tubular atrophy (Kolachalama *et al.* 2018, Mariani *et al.* 2018, Hermsen *et al.* 2019, Uchino *et al.* 2020, Bülow *et al.* 2021, Ginley *et al.* 2021, Jayapandian *et al.* 2021). Specialized work includes algorithms for locating and assessing glomeruli, stratification systems for IgA nephropathy (IgAN), and multiclass segmentation (Chen *et al.* 2019, Ligabue *et al.* 2020, Zeng *et al.* 2020, Bouteldja *et al.* 2021, Marechal *et al.* 2022, Yi *et al.* 2022). This rapid growing research is automating kidney pathology diagnosis, with implications for digital pathology applications. Supplementary Table S8 provided a summary of their datasets and applied methods to these studies.

Currently, most datasets of AI-based renal pathology are from adults and have not been validated on pediatric cohorts. The unique developmental characteristics of pediatric kidney structures and age-specific nephropathological conditions necessitate targeting and validating these AI algorithms for children. To address the challenges of rapid and accurate kidney pathological diagnosis in children CKD with limited numbers of nephropathologists, we established a pediatric kidney diseases database of a substantial number of annotations for network training. Based on the database, we developed an assistive diagnostic system for the segmentation and classification of kidney tissue structure, which we have named the AI-based Pediatric Kidney Diagnosis network (APKD). We compared the six current state-of-the-art network models, including newly released networks (Han *et al.* 2017, He *et al.* 2017, Cai and Vasconcelos 2019, Fang *et al.* 2021, Qiao *et al.* 2021). Supplementary Sections SI and SII provided the introduction of the models and comparison of their performance. After selecting the optimal network model (SCNet), we integrated it into the APKD framework. Thereafter, we trained this optimal AI model on a copiously annotated library of nephropathological WSI. We validated the trained model on segmentation and quantification of a set of comprehensive pathological features in WSIs of pediatric renal biopsies and validated the model using pediatric patient samples who were diagnosed with various kidney diseases.

## 2 Materials and methods

### 2.1 Patient cohort recruitment

**Figure 1.**
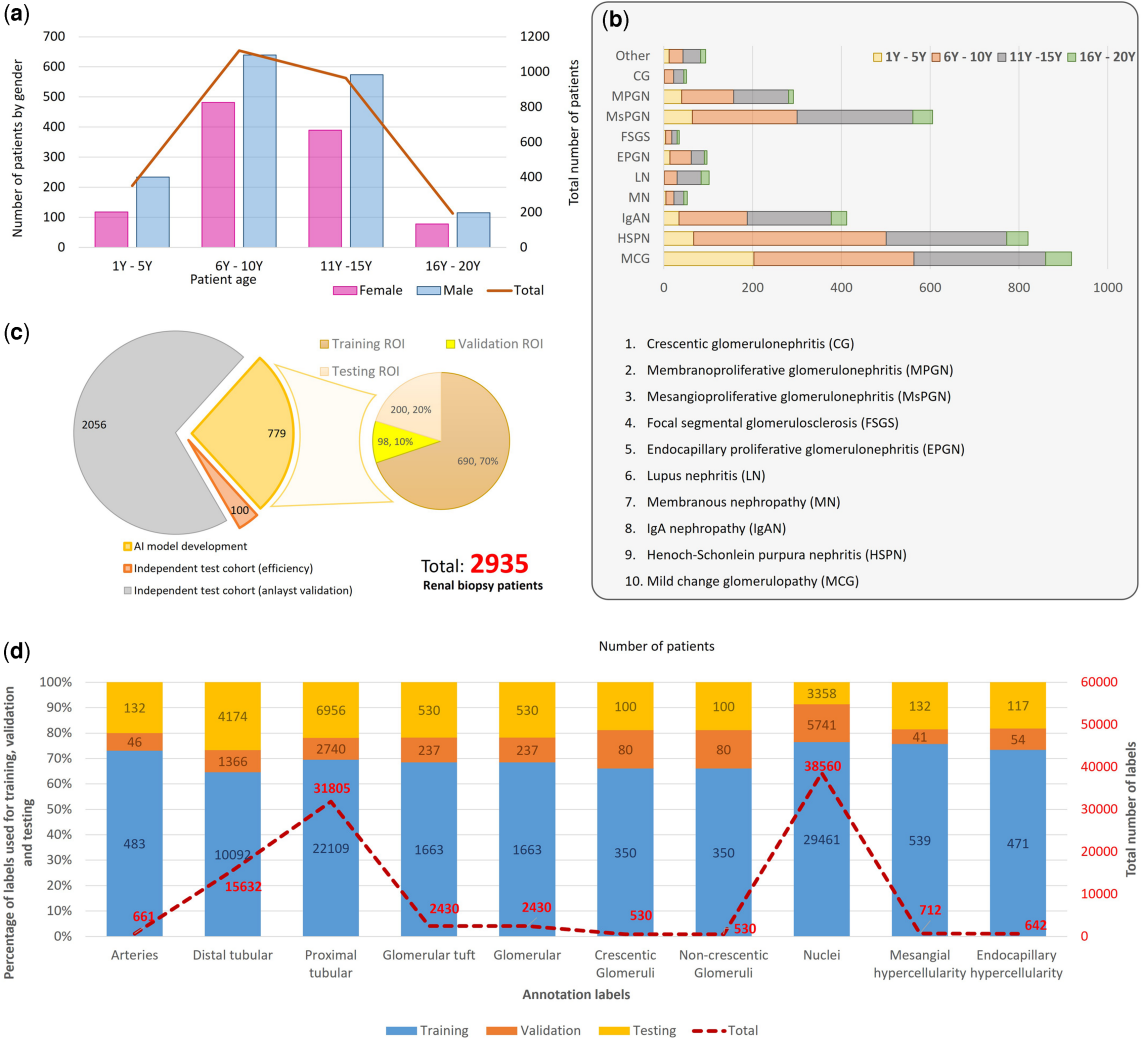
Profile of the recruited cohort and database statistics.  (a) A summary pediatric patient number, age, and male-to-female ratio. (b) Number of pediatric patient samples who were diagnosed with various kidney diseases. (c) The number of renal biopsy sections used to optimize AI development for training, validation, testing, and independent testing cohorts. Left pie chart: patient (biopsy) level; right pie chart: region of interest (ROI) level. (d) The number of annotations for kidney structures, crescent/non-crescentic glomerulus, nuclei, mesangial hypercellularity, and endocapillary hypercellularity used to perform training, testing, and validation.

The Department of Pathology, Children’s Hospital Affiliated to Zhejiang University School of Medicine, collected 2935 renal biopsy specimens of children diagnosed with kidney disease from 2009 to 2019 and stained with hematoxylin and eosin (H&E) staining and periodic acid-Schiff (PAS) staining, respectively. The requirements to obtain informed consent were waived by the Ethic Review Committee of Children’s Hospital of Zhejiang University School of Medicine (IRB2020-IRB-086), and all methods were carried out in accordance with relevant guidelines and regulations. Pediatric kidney diseases in the younger age group are mainly characterized by congenital anomalies of the kidney and urinary tract, or hereditary kidney diseases, so renal biopsy is not common in 1- to 5-year-old group and presents more commonly in boys (1562, 59.39%) than in girls (1068, 40.61%), typically between 6 and 15 years old, as illustrated by our data in Fig. 1a. In our sample collection, the children with kidney disease who underwent renal biopsy are mostly diagnosed as mild change glomerulopathy, Henoch-Schonlein purpura nephritis (HSPN), mesangial proliferative glomerulonephritis (MsPGN), and IgAN, as shown in Fig. 1b.

### 2.2 Sample digitalization and preparation

The renal biopsy tissues were scanned using a high-throughput KF-PRO-400 scanner at 10× (1 µm/pixel) and 40× (0.25 µm/pixel) magnifications. As our specimens were collected between 2009 and 2019, the quality of some samples degraded, such as discoloration and unclear nuclear staining. To obtain high-quality image data for AI training, we manually assessed image quality and excluded faded or unrecognizable tissue from the study. The number of patients with renal biopsy sections used for AI learning and development was 2935. Specifically, 779 patients’ kidney biopsy sections were used for AI model optimization and development, 100 patients for an independent testing cohort of efficiency performance, and 2056 patients for a separate, independent testing cohort of clinical validation. For AI model development patient cohort, there are 988 region of interest (ROI) extracted from 779 biopsy sections and split to training (690), validation (98), and testing (200) set with a ratio of 7:1:2 (see Fig. 1c). The ROI area was the region selected by pathologists on the WSI, and it typically must include glomeruli. The pathologists then annotated different kidney structures within the ROIs. All patients have paired sections of both H&E and PAS, while the 100-patient cohort in the efficiency independent test only has H&E slides. In addition, we accelerated the creation of nuclei annotations by obtaining them from a publicly available dataset (https://www.kaggle.com/c/data-science-bowl-2018/data). It contained many segmented cell nuclei images acquired under different conditions, including a range of cell type, magnification, and imaging modalities. In this study, a total of 856 nuclei images and their associated nuclei annotations were downloaded from the dataset named “data-science-bowl-2018.”

### 2.3 Data annotation scheme and strategies

Annotation of the image data is often the bottleneck of AI training as it can be expensive and time consuming. Three junior nephropathologists used the online kidney annotation platform to generate annotations and a senior nephropathologist reviewed the annotations. The annotation labels include arteries, distal tubule, proximal tubule, glomeruli with crescents, glomeruli without crescents, glomerular tuft, mesangial hypercellularity, and endocapillary hypercellularity, as shown in Fig. 1d.

**Figure 2.**
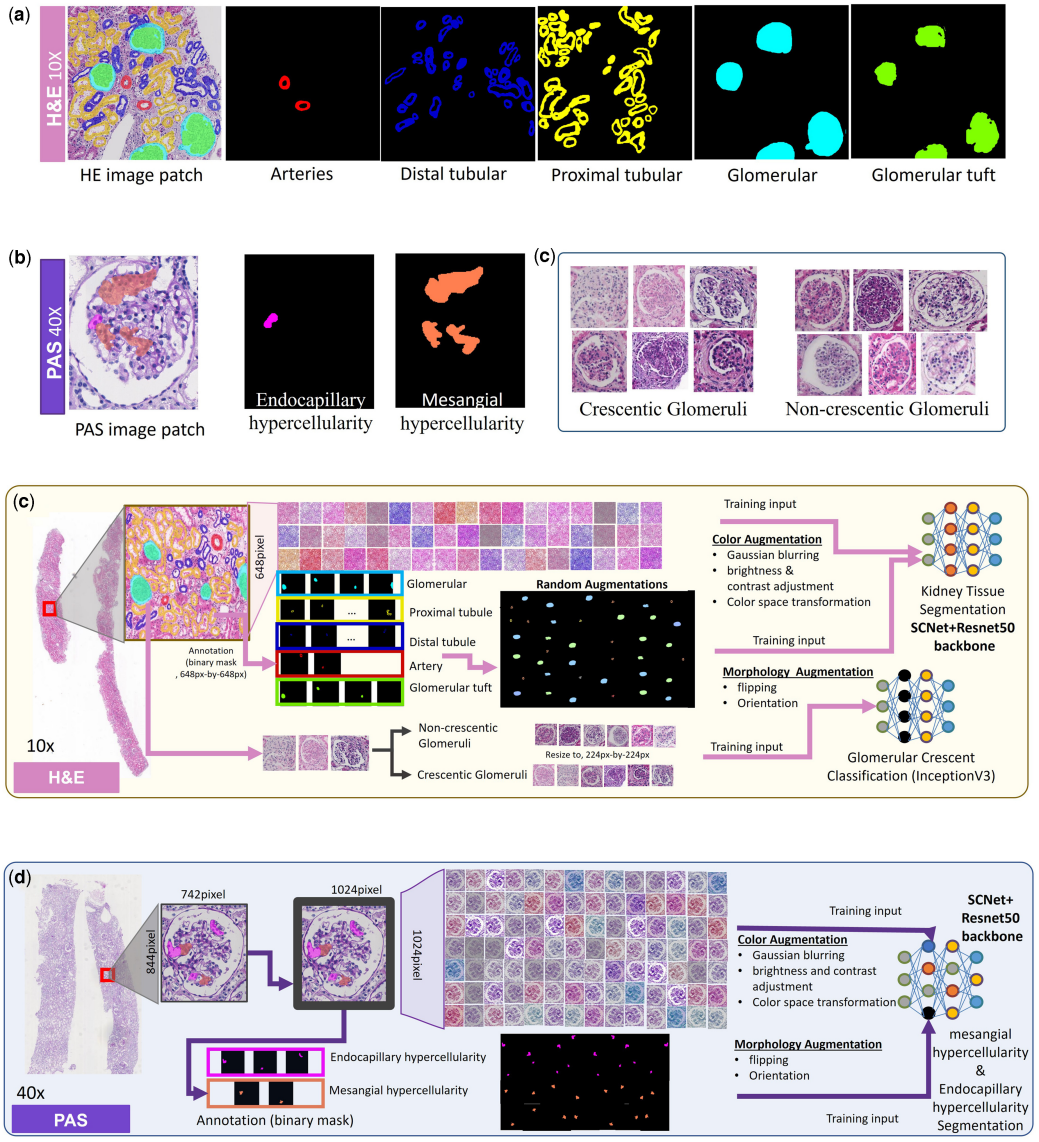
Annotation of the data and image argumentation for the training of APKD. (a) Annotations of various kidney tissues manually drawn by nephropathologists on 10× H&E images. (b) Annotations of endocapillary hypercellularity and mesangial hypercellularity manually drawn by nephropathologists on 40× PAS images. (c) Identification of glomeruli with and without crescents by nephropathologists. (d) Random augmentations such as orientation, flip, and color to train a SCNet model on 10× H&E images. (e) Random augmentations such as orientation, flip, and color to train a SCNet model on 40× PAS images.

Image patches of pixel size 648×648 (at a 10× magnification, 1 µm/pixel) which contain at least one glomerulus is cropped from each H&E-stained image. Glomeruli, glomerular tuft, proximal tubules, distal tubules, and arteries in this region are annotated as shown in Fig. 2a. The glomerular crescents and glomerular without crescents are identified and annotated separately (see Fig. 2c). Mesangial hypercellularity cells and endocapillary hypercellularity cells are annotated in PAS-stained images (at a 40× magnification, 0.25 µm/pixel) as illustrated in Fig. 2b since they are not recognizable in H&E-stained images. During our annotation of each selected glomerulus, we defined areas of hypercellularity (Chagas *et al.* 2020), as at least four mesangial cells aggregated in the mesangial area (mesangial hypercellularity) and at least two endothelial cells aggregated in capillary lumen area (endocapillary hypercellularity).

These image patches and their annotations were reviewed by a senior nephropathologist to ensure that the quality of the annotations was sufficient for the development of the APKD. Subsequently, when the average precision (AP, see Equation 1 in Section 2) of the AI detection model for kidney structure reached 77% from a total of 613 validated images with annotations, we then deployed a preliminary AI detection model to speed up the annotation review process. During the annotation review process, annotations drawn by the junior nephrologist were matched with the detection of APKD. We calculated mAP (mean value of AP of each class) for all H&E and PAS images separately, and a senior nephrologist reviewed and revised the annotations in images having the lowest 20% mAP. Hence, a total 93 932 histological structure annotations in the kidney have been manually annotated by three nephropathologists, and a total of 38 560 nuclear annotations were obtained from data-science-bowl-2018, as illustrated in Fig. 1d.

### 2.4 Model optimization

We developed and performed APKD training using the PyCharm integrated development environment (https://www.jetbrains.com/pycharm/), CUDA v10.2, CUDNN v7.6.5, and Pytorch v1.9.0 (https://pytorch.org/) running on a Ubuntu16.04 x64 operating system. The computer specifications used to develop and train AI algorithms are RAM: 32GB, CPU: Intel(R) Xeon(R) CPU E5-2643 v3 @ 3.40 GHz, and GPU: NVIDIA RTX 2080Ti *4.

In our APKD training, we use softmax cross-entropy as the loss function, a batch size of 16, and the Adam optimizer with a learning rate of 0.001. To avoid overfitting with limited data, we employ early stopping by monitoring the validation set. During training, the model’s weights are updated using the training set, while the validation set is used for inference. The average loss is tracked for both sets, with the model considered optimal when the validation set’s average loss reaches its minimum, which typically occurs after 24 training epochs. The model parameters yielding the lowest average validation mAP are saved post-training.

### 2.5 Framework of AI training and implementation

**Figure 3.**
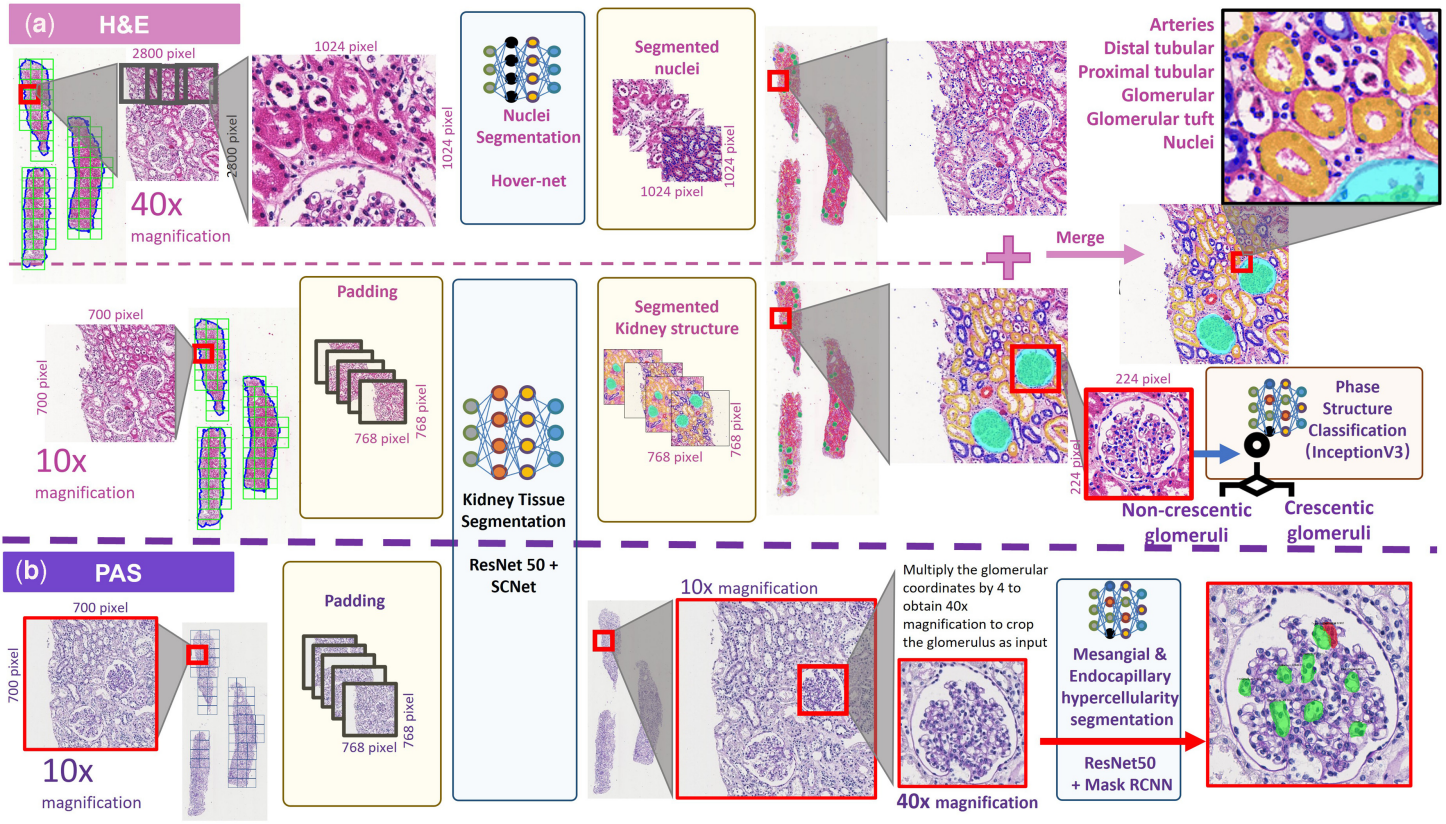
Overview of whole slide image detection and segmentation pipeline. (a) Nuclei detection and kidney tissue structure segmentation processes were performed using APKD based on 40× magnification H&E images and 10× magnification H&E images, respectively. (b) Endocapillary hypercellularity (indicated in green region) and mesangial hypercellularity (indicated in red region) detection process were performed using APKD based on 40× magnification PAS images.

An overview of our APKD development for multi-type automatic kidney tissue detection and segmentation is shown in Fig. 3. The proposed framework can be divided into four parts: (1) kidney structure segmentation module, (2) nuclei segmentation module, (3) glomerular crescents classification module, and (4) mesangial/endothelium region segmentation module. These four modules can be further split into two categories: the segmentation phase (Modules 1, 2, and 4) and the classification phase (Module 3), which are trained using different network structures. As shown in Fig. 3, digital H&E and PAS-stained renal biopsy WSIs are used as input, and the nucleus and kidney structures are segmented using H&E-stained images with 40× magnification and 10× magnification, respectively. At the same time, we use the PAS WSIs with 40× magnification to segment the mesangial and endothelium regions. Next, the glomerulus patches segmented from 10× H&E WSI are further classified as non-crescentic glomerulus or crescentic glomerulus.

#### 2.5.1 Modules 1 and 2: kidney structure segmentation module and cell nuclei segmentation module

For kidney structure segmentation, we first applied Otsu thresholding to remove the background from the renal biopsy of H&E on WSI. This method speeds up the analysis process by extracting only image data from the targeted region. A sliding window method is applied to extract the image patches. The size of the window is *n*-by-*n*, and stride is 0.5×*n*. *n* is 700 pixels for 10× images and 2800 pixels for 40× images as illustrated in Fig. 3a. We then modify the image patch size to 768 pixels for 10× images and 1024 pixels for 40× images through padding and resizing. These 10× and 40× H&E-stained image patches were used as input to the trained APKD model of kidney structure segmentation (SCNet+ResNet 50 as backbone) and the nuclei segmentation (HoverNet), respectively. The segmented nucleus masks at 40× H&E WSI were resized by a factor of 1/4 to obtain the corresponding mask at 10× magnification. Then the nuclei mask was integrated with kidney structure mask at 10×, and nonmaximum suppression was applied with 0.5 IoU threshold on the whole slide images.

Through this process, the segmentation-bounding box and mask of the module consist of arteries, distal tubules, proximal tubules, glomeruli, glomerular tuft, and nuclei. These outputs are feasible for obtaining a large amount of quantitative information for diagnosis or prognosis from digital renal biopsy sections.

#### 2.5.2 Module 3: glomerular crescents classification module

The glomerular patches segmented by kidney structure segmentation module were cropped and served as input to the glomerular crescent classification module. Due to the different sizes of the glomerulus being cropped, the image patches were first resized to 224×224, and then input to the trained APKD model of glomerular crescents classification module as demonstrated in Fig. 3a. We compared four lightweight convolutional networks for this module, including Resnet18 (He *et al.* 2016), Vgg16 (Simonyan and Zisserman 2014), MobileNetV2 (Sandler *et al.* 2018), and InceptionV3 (Szegedy *et al.* 2016). The InceptionV3 outperforms the other three models and is hence used for the glomerular crescent classification module. The accuracy of each model can be found in Section 3.

#### 2.5.3 Mesangial/endothelium region segmentation module

Similar kidney structure segmentation process performed on H&E-stained WSI was applied to PAS-stained WSI for glomerular detection. First, we extracted 700×700 image patches by sliding windows in 10× PAS WSI and increased the image patch size to 768 pixels through a padding process. These patches were used as input to the trained model of kidney structure segmentation developed from H&E WSI because the color augmentation process can compensate for the color variation in PAS-stained image to segment the glomerulus. Subsequently, the detected glomerulus bounding box coordinates (x, y, w, h) were multiplied by 4 to obtain the corresponding coordinates at 40× magnification PAS-stained images as demonstrated in Fig. 3b. The glomerular patches were cropped from 40× PAS image and the endocapillary hypercellularity/mesangial hypercellularity region in each image patch was detected by the trained APKD model of endocapillary hypercellularity/mesangial hypercellularity segmentation using Mask RCNN with Resnet50 as the backbone. Finally, we integrated the results of all patches and performed nonmaximum suppression with the threshold of IoU = 0.5 on the whole slide images.

### 2.6 Evaluation metrics for detecting each kidney tissue, glomerular structure, and glomerulus counting efficiency

For the kidney structure detection model, we reported the average precision (AP) metric defined in PASCAL VOC 2010 (Everingham *et al.* 2010) to assess the model performance, as shown in Equation (1).

(1) $$\mathrm{AP}=\frac{1}{11}\sum_{r\in \left[0,0.1,\ldots ,1\right]}P_{\mathrm{interp}}(r),P_{\mathrm{interp}}\left(r\right)=\max_{\tilde{r}:\tilde{r}\geq r}p\left(\tilde{r}\right),$$

$$\mathrm{where}\;p\left(\tilde{r}\right)\;\mathrm{is}\;\mathrm{the}\;\mathrm{measured}\;\mathrm{precision}\;\mathrm{at}\;\mathrm{recall}\;\tilde{r}$$

We calculate AP50 using 11-point interpolation. First, we average a set of 11 equally spaced recall values [0, 0.1, 0.2, …, 1] and their corresponding precision to create a precision–recall curve. When computing precision, for a specific recall value r, precision is taken as the maximum value among all recalls ≥r. This ensures the precision–recall curve is monotonically decreasing, preventing fluctuations in the curve. AP_50_ means the IoU threshold is set at 0.5.

Commonly used evaluation metrics such as sensitivity [Equation (2)], specificity [Equation (3)], and precision [Equation (4)] were used as well.

(2) $$\begin{array}{cc} \mathrm{Recall}\left(\mathrm{Sensitivity}\right)=\;\frac{\mathrm{TP}}{\mathrm{TP}+\mathrm{FN}}, & \end{array}$$

(3) $$\begin{array}{cc} \;\mathrm{Specificity}=\;\frac{\mathrm{TN}}{\mathrm{TN}+\mathrm{FP}}, & \end{array}$$

(4) $$\begin{array}{cc} \mathrm{Precision}=\frac{\mathrm{TP}}{\mathrm{TP}+\mathrm{FP}}, & \end{array}$$

where TP represents true positive, TN the true negative, FP the false positive, and FN represents false negative. As for the crescentic glomeruli classification model, area under receiver operating characteristic curve (AUC) is used to assess the performance.

We compared the performance of nephropathologists and the trained model on counting glomeruli and conducted an experiment with 98 cases new WSI dataset. A senior nephropathologists counted the number of glomeruli on the glass slides under microscope and recorded the time spent. Meanwhile, the trained model was applied to the digitalized images of these cases to count the number of glomeruli, and the processing times were tracked.

### 2.7 Statistical analysis

The Spearman correlation coefficient and intra-class correlation coefficient (ICC) tests were employed to measure the correlation and agreement, respectively. A Pearson correlation value of 1 indicates the best correlation, and a *P*-value of <.05 is considered statistically significant. To test the consistency, we calculated the ICC and its corresponding 95% confidence interval (CI) to determine the coincidence between the number of glomeruli detected by the AI model and the observer counted manually. According to the procedure recommended by Koo and Li (2016), the appropriate ICC parameter selected for this reliability analysis is where the reliability of a single measurement has “absolute consistency” because there are two evaluators (manual and AI models). In addition, we used analysis of variance (ANOVA) test to compare the number of glomeruli across the age range of pediatric patients. All statistical analyses were performed in MATLAB 2014a (Mathworks Inc., USA) and its statistical toolbox, except the ANOVA test, performed by JASP (https://jasp-stats.org/), a free and open-source program for statistical analysis developed in the R language.

## 3 Results

### 3.1 Model optimization and detection performance of APKD

**Figure 4.**
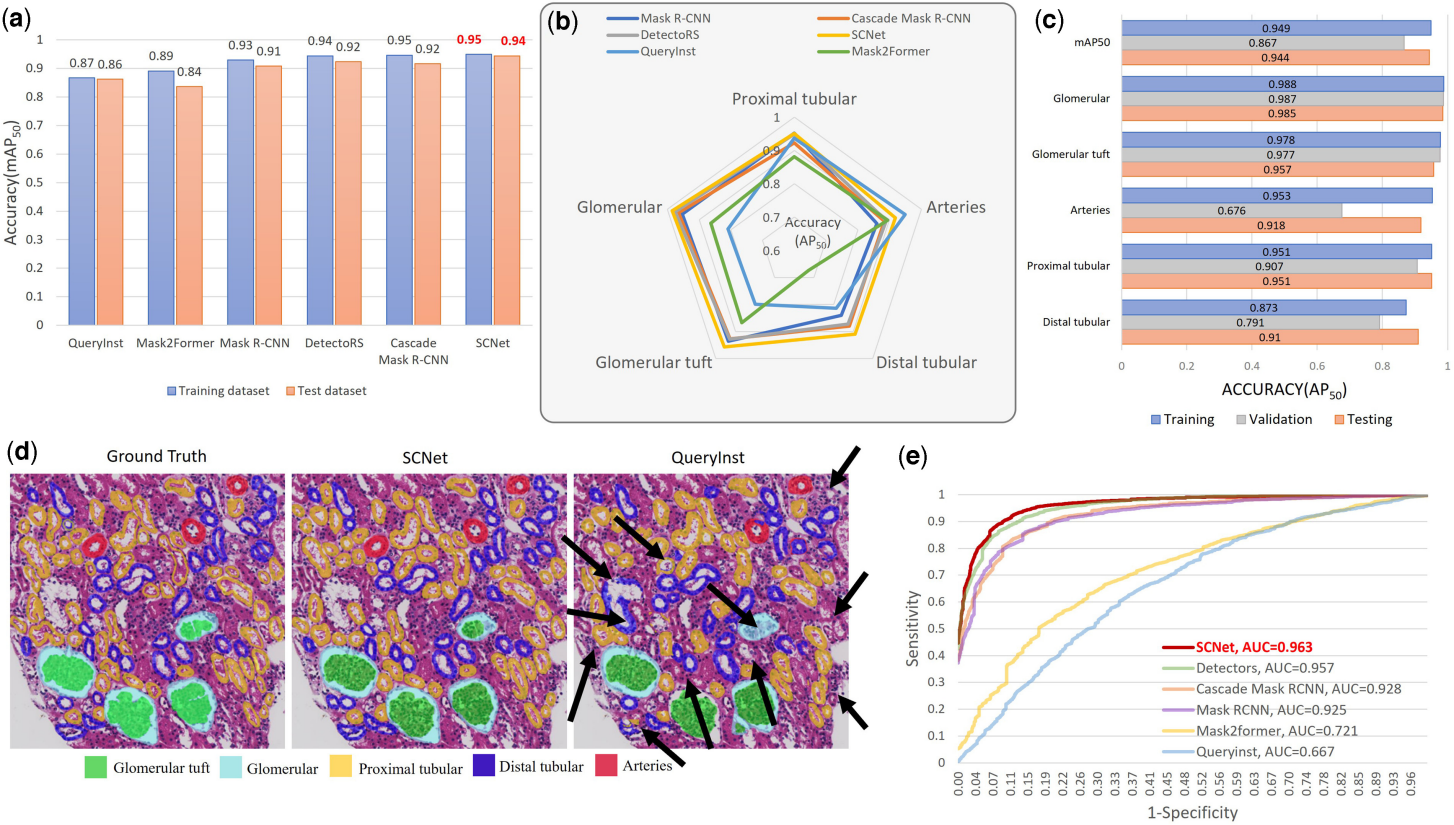
Model optimization of APKD. (a) Comparison of mAP_50_ of different models in training and testing set. (b) AP_50_ of different models for different classes in testing set. (c) Comparison of AP_50_ of SCNet for different classes in training, validation, and testing set. (d) Manual delineation (Ground Truth), compared to segmentation results generated by SCNet and QueryInst; arrows indicate undetected structures. (e) The mean of receiver operating characteristic (ROC) curve across all classes and the corresponding AUC of testing set for each model are compared.

The kidney structure detection model of the proposed APKD is optimized from six latest CNN models. The details of six models are provided in Supplementary Section SI and the optimization results are provided in Supplementary Section SII. All six models were based on the ResNet 50 backbone due to its success application in the field. We evaluated the accuracy (in terms of mAP50, which is the mean value of AP50 of each class) of six models in both training set and testing set as shown in Fig. 4a. SCNet with ResNet 50 as the backbone outperformed the other five models and achieved 0.95 and 0.94 mAP50 in training and testing set, respectively. The data clearly show that the SCNet achieved the optimal performance with intermediate computational efficiency which is shown in Supplementary Table S2. The average precision (AP50) of each class for six models in testing set is demonstrated in Fig. 4b. SCNet ranked first among all models in three classes (Glomerular, AP = 0.99; Glomerular tuft, AP = 0.96; and Distal tubular, AP = 0.91), and second in the other two (Proximal tubular, AP = 0.95; and Arteries, AP = 0.92). As illustrated in Fig. 4c, SCNet achieved more than 0.9 testing set average precision in all classes; in particular, the average precision was 0.99 for Glomerular, the most important structure. We also reported the mean of receiver operating characteristic (ROC) curve and AUC across all classes of testing set for each model, as shown in Fig. 4e. SCNet achieved high sensitivity and specificity with AUC of 0.963. The sensitivity, specificity, and AUC of SCNet in each class are shown in Supplementary Table S3.

Both mAP50 and AUC accuracy differed substantially between QueryInst and SCNet (0.86 versus 0.94 in mAP50, and 0.667 versus 0.963 in AUC). After interrogating the results of QueryInst and SCNet, we found at least 11 target misses in QueryInst detection compared to ground truth (indicated by black arrows, Fig. 4d), while SCNet clearly showed an almost perfect match with the ground truth.

To assess the speed of detection, a total of 200 image patches (648×648 pixel) were used to compare the detection time among six models. We observed a mean inference time of the six models for one image is about 0.3 s on a standard workstation (see Supplementary Table S4). Given the challenge for nephropathologists to continuously perform visual inspections of WSIs while maintaining a high degree of accuracy, the automatic detection of AI can thus effectively and quickly assist nephropathologists to identify diseased kidney tissue.

### 3.2 APKD detection performance and quantification of various types of kidney structures for assistive diagnosis

**Figure 5.**
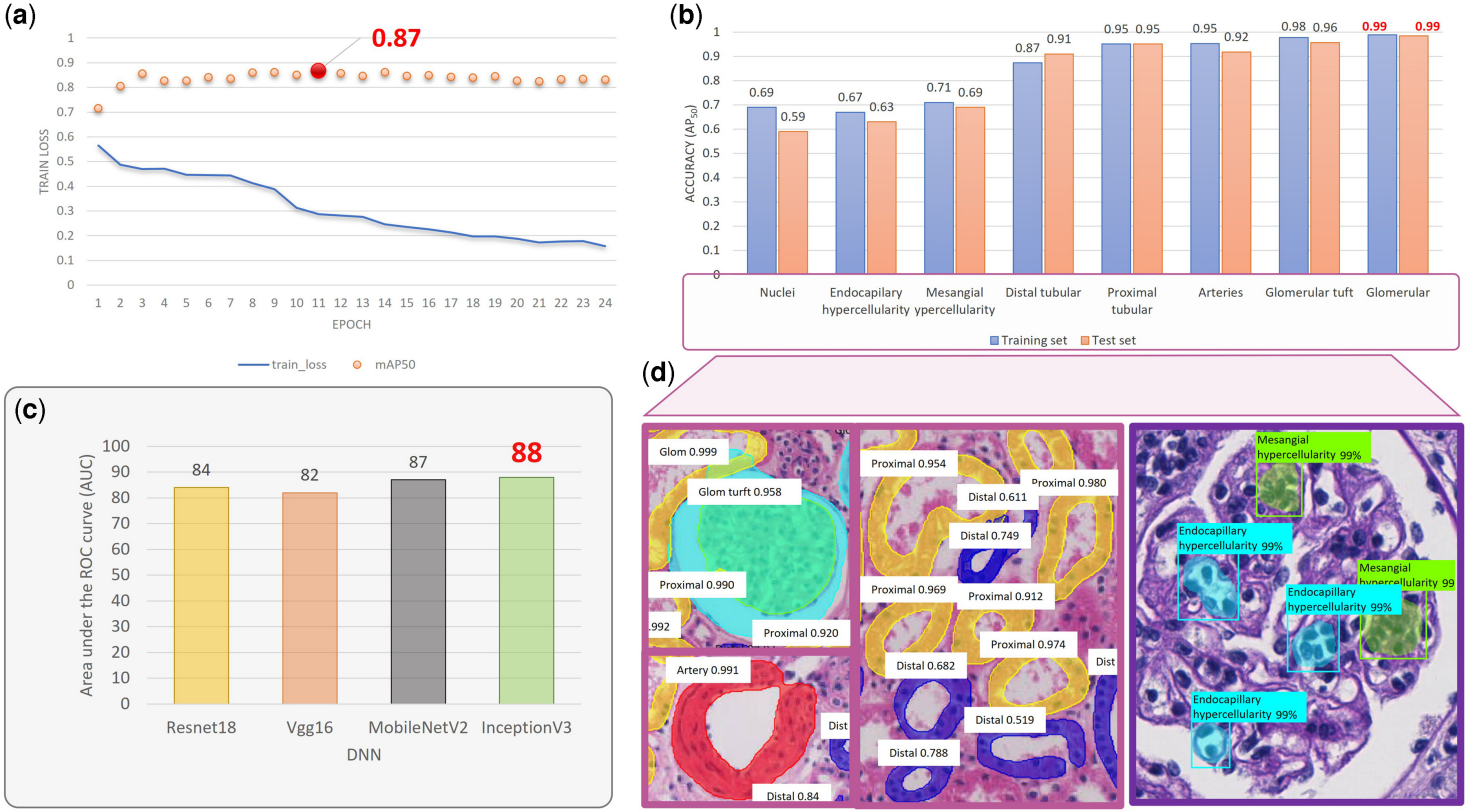
Quantitative analysis of APKD detection performance. (a) The loss function during training and the optimal mean Average Precision of the SCNet model is 87%. (b) APKD detection accuracy (AP_50_) for all kidney structures and nuclei in testing set. (c) Resnet18, VGG16, MobileNetV2, and InceptionV3 assessed crescentic glomerulonephritis detection accuracy by area under the receiver operating characteristic (ROC) curve. (d) Visualization results of APKD detection and segmentation for all kidney structures and nuclei.

To optimize our APKD model, we iterated the SCNet training process for 24 epochs until the mAP of the validation set no longer improved, with an optimized precision of 87%, as shown in Fig. 5a. The detection accuracy (AP50) for the testing set of APKD for each class ranged from 0.59 to 0.99 as in Fig. 5b and their corresponding representative segmentation results are illustrated in Fig. 5d. The number of glomeruli is a key parameter that nephrologists need to consider in the diagnostic process. As shown in Fig. 5b, the accuracy of APKD for glomerular detection reached 99%. Identifying the formation of glomerular crescent can help detect the progression of crescentic glomerulonephritis. Our classification and quantification of 12 related features, including area of glomerular crescent, length, and roundness, will serve as a powerful reference parameter for nephropathologists to give correct diagnoses. We compared the performance of four lightweight convolutional networks, Resnet18, Vgg16, MobileNetV2, and InceptionV3 in glomerular crescent classification, as demonstrated in Fig. 5c. The InceptionV3 model has an AUC of 88% on the testing set and clearly outperforms the others (Resnet18: 84%, Vgg16: 82%, MobileNetV2: 87%).

**Figure 6.**
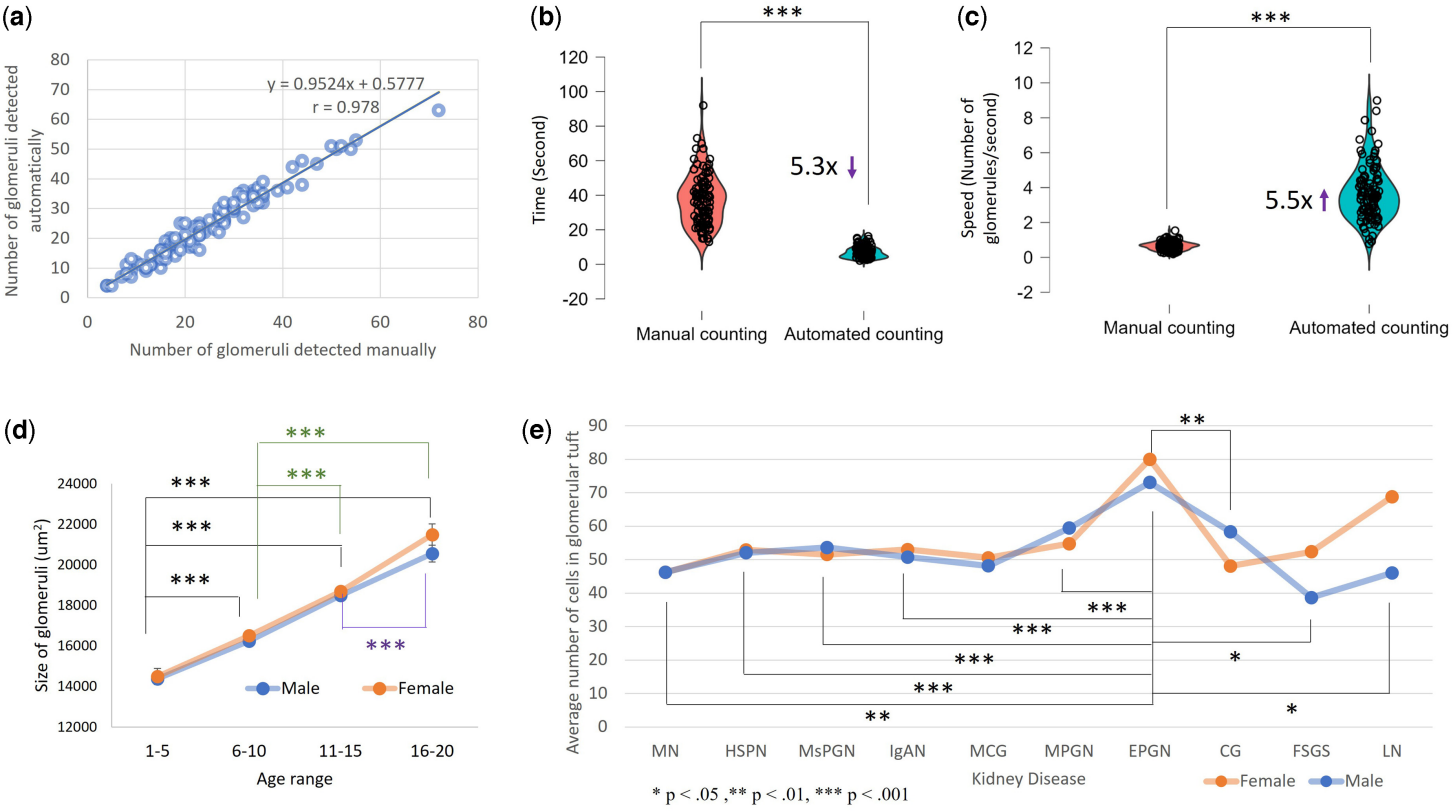
Consistency between manual detection and the AI model. (a) Correlation evaluation between manual glomeruli count and automatic glomeruli detection. (b) Comparison of time spent for manual glomeruli counting and automated glomeruli detection. (c) Speed evaluation between manual glomeruli count and automatic glomeruli detection. (d) Detected size of glomeruli by APKD in children of different age range and genders (***P < .001 regardless the gender). (e) Detected number of cells in glomeruli by APKD in children of different kidney disease and genders (***P < .001 and **P < .01 regardless of the gender).

We employed an independent testing cohort (1625, retrospective) for clinical analyst validation. Spearman correlation coefficient and ICC were used to measure the correlation and consistency between the number of glomeruli detected by the APKD model and the manual detection by experienced nephropathologists, showing a significant and strong correlation between the APKD model and manual detection by the nephropathologists (r = 0.9778, *P*<.001; see Fig. 6a). As an additional reliability test, we obtained the ICC to evaluate the consistency between manual glomerulus detection by the nephropathologists and the AI model. The result shows that the ICC using the APKD model is 0.9765 (95% CI=0.9647–0.9843), showing strong correlation. We validated the speed of the automated method versus the manual method for counting glomeruli on 100 H&E kidney digital pathology images as an additional independent testing cohort. Nephropathologists spent an average of 37 s per WSI, while the average processing time of the model was only 7 s, as illustrated in Fig. 6b. In general, the model is approximately 5.5 times faster than nephropathologists in counting glomeruli (counts of glomeruli/sec), as shown in Fig. 6c.

In the early days of pathological quantification, Nyengaard and Bendtsen (2010) reported age-related studies on the number and size of glomeruli and reached a consensus in this field. However, the quantitative information about the relationship between the number and size of glomeruli and increased age is scant, especially in child cohorts. Therefore, we analyzed the area of glomeruli detected in a cohort of children aged 1–5, 6–10, 11–15, and 16–20 years with the assistance of APKD and their gender using ANOVA testing, as shown in Supplementary Table S5. It revealed a significant increase in glomeruli areas from ages 1–5 until 16–20 years for both females and males (*P*<.001, *post hoc* by Tukey with correction for multiple comparison for the four families), as illustrated in Fig. 6d.

The total number of nucleated cells in the glomerular tuft has been quantified in various kidney diseases, and we have observed that the number of nucleated cells in the glomerular tuft of endocapillary proliferative glomerulonephritis (EPGN) is significantly higher than that of other diseases (*P*<.001 by the ANOVA test, *post hoc* by Tukey with correction for multiple comparison for the 10 families; see Fig. 6e and Supplementary Table S6).

## 4 Conclusion and discussion

The focus of this research was to develop and apply DL solutions to help nephropathologists rapidly identify and detect kidney structures for quantification. We also derived tissue morphological features from segmentation calculations to enable biopsy interpretation. The proposed framework and applied DL network we developed helps to pave the way for future development of kidney tissue assays by providing a network optimized for, trained on, and validated by a cohort of highly annotated pediatric patient samples. The trends in pathomorphological features we have shown for pediatric kidney disease subtypes may also help to establish a baseline from which to assess kidney disease prognosis or predict treatment response.

The comprehensive set of pathomorphological features detected and quantified through our framework can help nephropathologists quickly understand the organization of structural indexes in renal tissue, including the number of glomeruli, glomerular volume, cells in the glomerulus, area of the mesangial matrix, formation of crescents, and tubules. Such a solution has the potential for facilitating early diagnosis, guiding therapeutic strategy, and prognosticating the evolution of glomerular diseases for pediatric kidney disease. Another potential application is that the annotations generated by APKD will be superimposed on WSI as a navigator to effectively assist the pathological examination of each ROI. Thus, this platform helps reduce the tedious and repetitive workload of nephropathologists.

Rapidly progressive glomerulonephritis is a kidney disease whose chief clinical feature is a rapid decline of at least 50% in the glomerular filtration rate within a short period, from a few days to three months. The main pathological finding is extensive glomerular crescent formation. The common pathological feature of crescents is the proliferation of parietal epithelial cells. Our glomerular crescents classification module and 58 comprehensive pathomorphological features can help to detect crescent for early diagnosis (see Supplementary Table S7). Clinically, crescents are described as cellular, cellular fibrous crescent, and fibrous crescent, which are more accurate indicators for the prognosis and response to treatment. This APKD cannot distinguish the crescents with detail now, and further work is needed to encompass this complexity. Our model trained on samples across the pediatric age range also allowed us to show a main effect from age after automatically calculating the number and size of glomeruli, indicating that the size of the glomerulus increases linearly with age during this period (see Fig. 6d and Supplementary Table S5). Our quantitative results are consistent with earlier reports in adults and extend these findings to child cohorts (Nyengaard and Bendtsen 2010).

The identification and classification of glomerular lesions are fundamental steps toward the diagnosis of most kidney diseases. Of particular importance is glomerular hypercellularity, a lesion defined by an increase in the number of cells in the glomeruli and frequently found in pediatric kidney disease, including IgAN, HSPN, and MsPGN. The presence and abundance of glomerular hypercellularity are closely related to the diagnosis, severity, and prognosis of disease. Our study can quantify the number of nucleated cells in the glomeruli of children with kidney disease (see Fig. 6e and Supplementary Table S6). Future implementations will need to distinguish features of these cell types (e.g. podocytes, mesangial cell, and endothelial cell) by AI, which may serve as important pathomorphological features in future kidney AI studies.

Many previous studies have only aimed to identify glomerulosclerosis (Ginley *et al.* 2019, 2021, Altini *et al.* 2020), a few structures (e.g. renal tubules (Bevilacqua *et al.* 2017), interstitial fibrosis, and renal tubular atrophy), or pathological features of specific diseases, such as diabetic nephropathy, IgAN (Zeng *et al.* 2020), or lupus nephritis. Furthermore, in most studies, the size of the dataset was rather small (*n* < 100 WSI). The results of our study show that our method, supported by big data (*n* > 1000 WSI), can accurately detect multiple types of kidney structures and cells in different kidney diseases. Furthermore, as indicated by the kidney studies mentioned above, the vast majority of AI nephrology studies to date have focused on adults rather than children with kidney disease. As the number of cases in children increases (Shi *et al.* 2021), more attention needs to be paid to the unique pathology of kidney disease. In addition, AI is also necessary for the development of kidney disease, especially the comprehensive detection and quantification of various structures in renal biopsies to help nephropathologists rapidly quantify and analyze tissue samples of all age groups. Our results in a pediatric dataset suggest that AI is suitable for use in children with kidney disease, especially for identification of glomeruli, with the ability to accurately count glomeruli of different sizes and at different ages while achieving consistent diagnoses with those of nephropathologists.

However, our study has limitations. First, our datasets used in research are not large enough and are from a single Chinese children’s hospital; the applicability of the results to other institutions remains to be verified. Second, our dataset was limited to H&E and PAS staining. Third, the glomerulus contains three resident cell types: mesangial, endothelial, and podocytes. Although podocyte loss or podocytopenia is important to assess the progression of glomerular disease, our work has yet to segment podocytes. To address these limitations, we are currently collecting a multicenter kidney disease dataset from Chinese hospitals. These datasets will include several stains used to diagnose kidney disease, such as H&E, PAS, Silver, and Trichrome. Given the rapid pace of advancement in AI models, we will also incorporate the latest models, such as the Swin-transformer-base network model (Liu *et al.* 2021), to enhance the detection accuracy and generalizability of our model, while comparing the detection efficiency and accuracy performance of the current models.

In conclusion, our automated APKD extracts detailed quantitative features to interpret renal biopsies and assist nephropathologists to make rapid assessments. In future work, we will combine the path-morphology identified by APKD with clinical features such as medical history and laboratory tests extracted through AI and larger, multicenter datasets to enhance the accuracy and broaden the applicability of APKD for diagnosis of pediatric kidney disease.

## Supplementary Material

btad740_Supplementary_Data

## Data Availability

A subset of the annotations created by renal pathologists for the development and verification of the AI models is published for reference purposes at https://github.com/ChunyueFeng/Kidney-DataSet. However, due to sample information protection, patient privacy protection, and medical institutional data regulatory policies in China, the full data for the development and verification of the proposed method are not publicly available, but C.F. (springmoonfeng@163.com) can be contacted directly.
