# Supplementary material for: Artificial intelligence-assisted quantification and assessment of whole slide images for pediatric kidney disease diagnosis
Source: Bioinformatics. 2023 Dec 7;40(1):btad740. doi: 10.1093/bioinformatics/btad740 (PMC10796177; doi:10.1093/bioinformatics/btad740)
Supplement: btad740_Supplementary_Data [file btad740_supplementary_data.docx]

**Artificial intelligence-assisted quantification and assessment of whole slide images for pediatric kidney disease diagnosis**

**Supplementary Material**

[**Artificial intelligence-assisted quantification and assessment of whole slide images for pediatric kidney disease diagnosis** 1](#_Toc150718964)

[**Section I: Model introduction** 2](#_Toc150718965)

[**Model 1: ResNet 50+Mask R-CNN** 2](#_Toc150718966)

[**Model 2: ResNet 50+Cascade Mask R-CNN** 2](#_Toc150718967)

[**Model 3: ResNet 50+DetectoRS** 3](#_Toc150718968)

[**Model 4: ResNet 50+SCNet** 3](#_Toc150718969)

[**Model 5: ResNet 50+QueryInst** 4](#_Toc150718970)

[**Model 6: ResNet 50+Mask2Former** 5](#_Toc150718971)

[**Data Preparation and Augmentation** 5](#_Toc150718972)

[**Data Distribution and Experimental Setup** 6](#_Toc150718973)

[**Section II: Test results across different models** 6](#_Toc150718974)

[**Model Performance** 6](#_Toc150718975)

[**Inference Speed and Inference Result** 7](#_Toc150718976)

[**Correlation and Statistical Analysis** 8](#_Toc150718977)

[**Section III: Common pathological features extracted using APKD** 9](#_Toc150718978)

[**Section IV: Summary of recent publication on deep learning based kidney pathological diagnosis** 10](#_Toc150718979)

[**Reference** 12](#_Toc150718980)

# **Section I: Model introduction**

We trained six models for kidney structure segmentation using ResNet 50 [1] as backbone, their structures are as follows.

**Model 1: ResNet 50+Mask R-CNN**

Mask R-CNN [2] is a flexible model which is able to perform object classification, object detection, semantic segmentation, instance segmentation, and gesture recognition tasks. The instance segmentation is achieved by adding a mask estimation branch to the existing object classification and bounding box regression branch. The estimation of object mask, which is equivalent to a two-class semantic segmentation, is performed in each region of interest (ROI). The object detection and segmentation branch and the object classification and bounding box regression branch are parallelly executed. The idea of Mask R-CNN is to add a fully convolutional network (FCN) to the Faster R-CNN model to generate the mask branch, i.e., Mask R-CNN = Faster R-CNN + FCN, to be more specific, Mask R-CNN = RPN + ROIAlign + Fast R-CNN + FCN. Fig. S1 demonstrate the structure of Mask-RCNN.


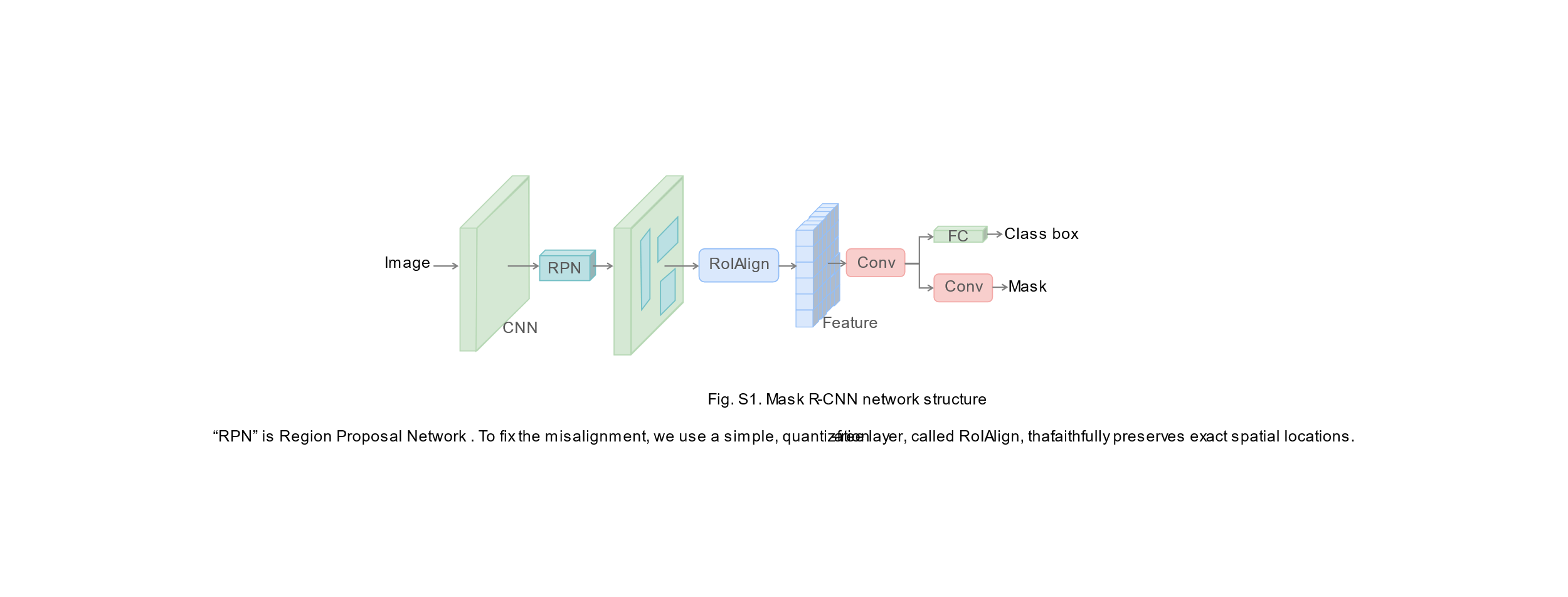


**Fig. S1. Mask R-CNN network structure** Mask R-CNN uses the Region Proposal Network (RPN) and a simple, quantization-free layer, called RoIAlign to ensure precise object localization. The network includes fully connected layers for class prediction and bounding box regression. Additionally, it features a mask prediction branch for pixel-level object segmentation.

**Model 2: ResNet 50+Cascade Mask R-CNN**

Figure 2 shows architectures of Faster RCNN and three cascade strategies. the In Faster R-CNN (Fig. S2a), most proposals generated by the Region Proposal Network (RPN) lack high quality. Fig. S2b illustrates that the shared H positions in Iterative BBox create numerous outliers in the later two stages. Using shared H positions fails to accommodate the variations in detector inputs. And the Integral Loss (Fig. S2c) shares the pooling step, involving only one stage, but there are three distinct H positions, each corresponding to different IoU thresholds. In the first stage, the distribution of input IoUs is highly uneven, with very few high-threshold proposals. Consequently, detectors responsible for high-threshold values tend to overfit easily.

The structure of Cascade R-CNN [3] , as demonstrated in Fig.S2d, consists of several stage of detection networks with different intersection over union (IoU) thresholds cascaded to the Faster R-CNN, the thresholds are increasingly ordered. The stage with lower IoU threshold avoids overfitting problem due to insufficient positive samples. The output of low IoU threshold detector is the input of high IoU threshold detector for higher accuracy. Cascading regression is used in Cascade R-CNN as a resampling scheme to progressively increase the IoU of proposal, so that every stage has sufficient positive samples to avoid overfitting problem.

Cascade Mask R-CNN enhances Cascade R-CNN for instance segmentation by incorporating a mask head into the cascade structure. It introduces multiple detection branches and offers three strategies for mask prediction. These strategies include adding a single mask prediction head at either the first or last stage of the cascade and adding a segmentation branch to each cascade stage. During inference, all three strategies predict segmentation masks on patches generated by the final object detection stage, optimizing mask prediction diversity.


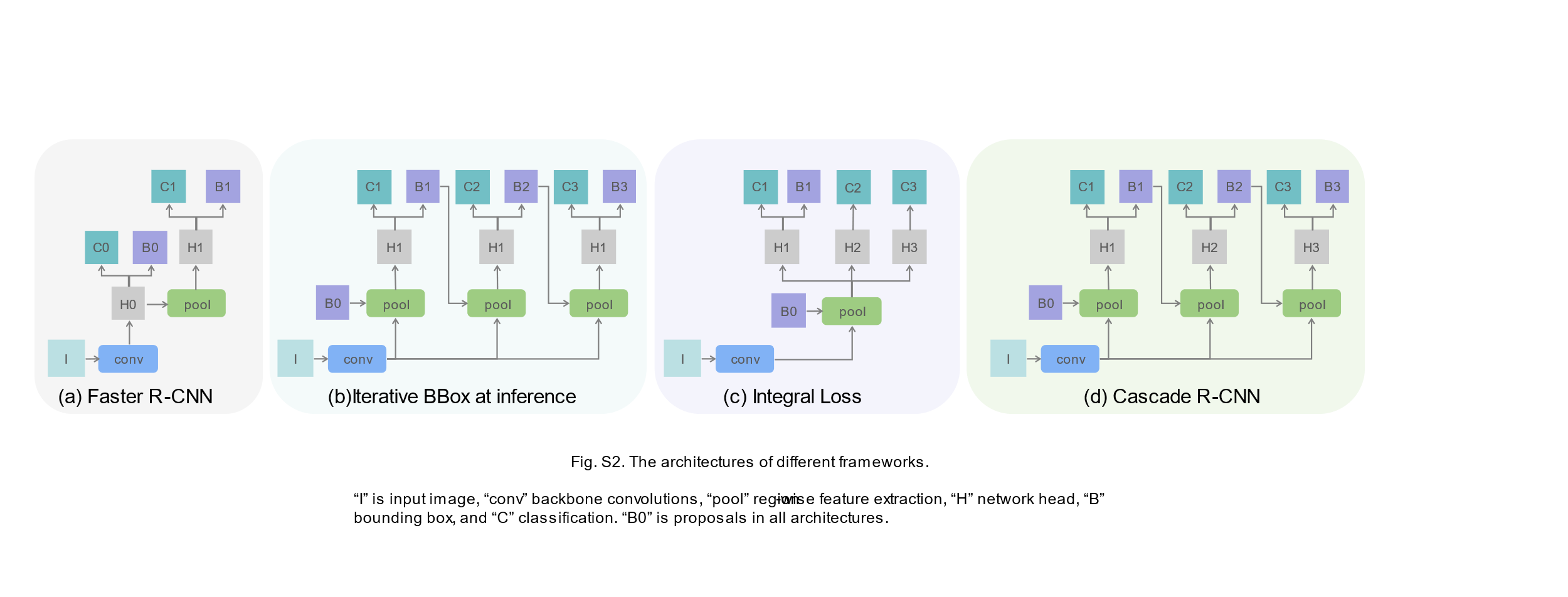


**Fig. S2. Comparison of the Cascade Structure with Similar Architectures** “I”: input image, “conv” backbone convolutions, “pool”: region-wise feature extraction, “H”: network head, “B”: bounding box, “C”: classification. “B0”: proposals in all architectures.

**Model 3: ResNet 50+DetectoRS**

The idea of looking and thinking twice was used in many object detectors and achieved good performance. The author of DetectoRS [4] explored this mechanism in the backbone design for object detection.

At the macro level, recursive feature pyramid (RFP) was proposed, which incorporates extra feedback connections from feature pyramid networks (FPN) into the bottom-up backbone layers. At the micro level, switchable atrous convolution was proposed, which convolves the features with different atrous rates and gathers the results using switch functions. DetectoRS is obtained by combination of these two structures, which significantly improves the performances of object detection.


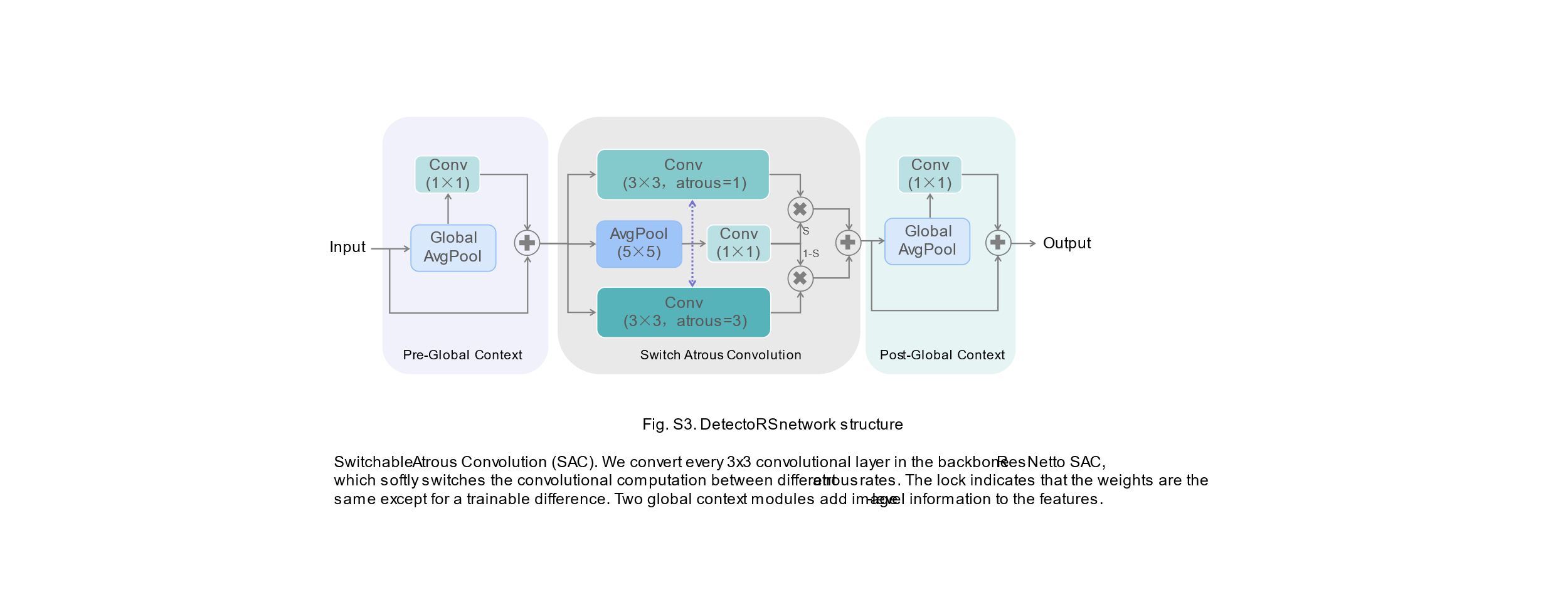


**Fig. S3. DetectoRS network structure** We convert every 3x3 convolutional layer in the backbone ResNet to Switchable Atrous Convolution(SAC),which softly switches the convolutional computation between different atrous rates. The lock indicates that the weights are the same except for a trainable difference. Two global context modules add image-level information to the features.

**Model 4: ResNet 50+SCNet**

Sample Consistency Network (SCNet) [5], aimed at maintaining consistency between the Intersection over Union (IoU) distribution of samples during training and inference. SCNet achieves this by incorporating feature relay and leveraging global contextual information, strengthening the interplay between classification, detection, and segmentation tasks. Notably, when compared to Cascade Mask R-CNN, SCNet demonstrates enhancements in both bounding box and mask prediction Average Precision (AP), along with improved processing speed.


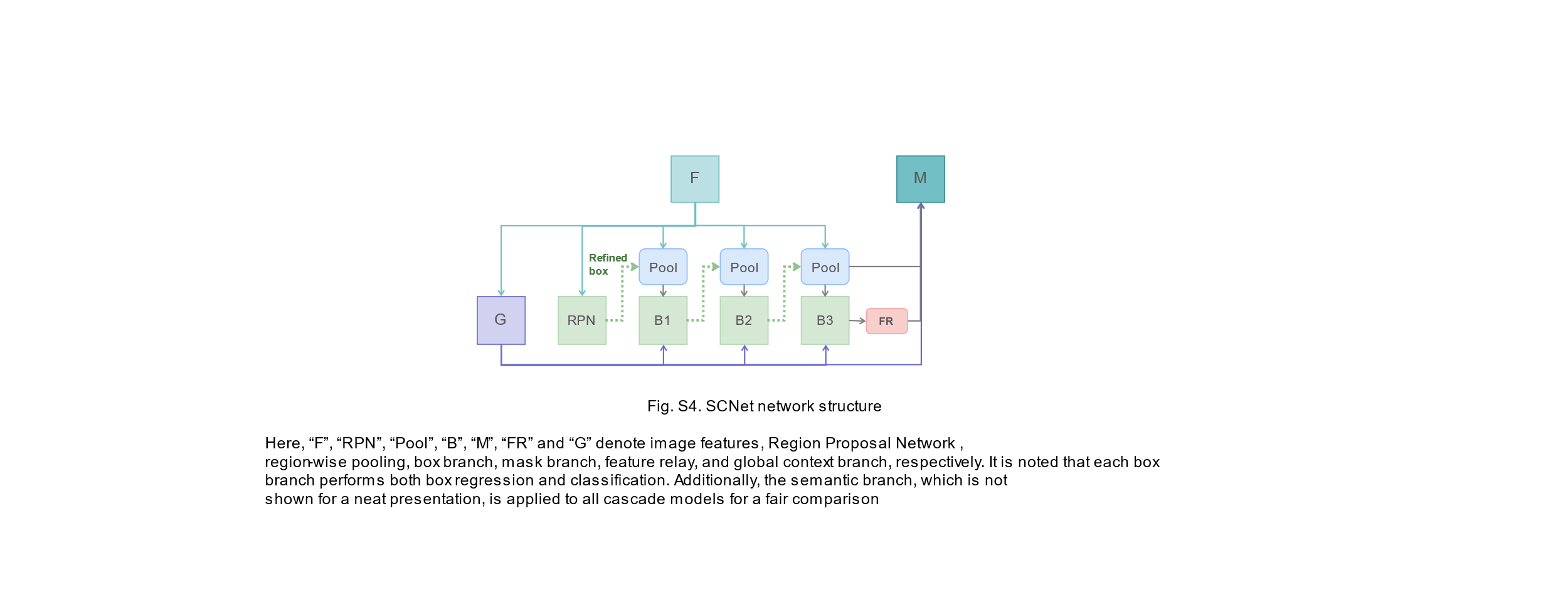


**Fig. S4. SCNet network structure** “F”, “RPN”, “Pool”, “B”, “M”, “FR” and “G” denote image features, Region Proposal Network, region-wise pooling, box branch, mask branch, feature relay, and global context branch, respectively. It is noted that each box branch performs both box regression and classification. Additionally, the semantic branch, which is not shown for a neat presentation, is applied to all cascade models for a fair comparison.

**Model 5: ResNet 50+QueryInst**

QueryInst [6] is an instance segmentation technique based on queries and dynamic mask heads with parallel supervision. QueryInst capitalizes on the inherent one-to-one correspondence in object queries between different stages and between mask ROI features and object queries within the same stage. This innovative approach eliminates the need for explicit multi-stage mask head connections and resolves issues related to proposal distribution inconsistencies commonly found in non-query-based multi-stage instance segmentation methods.


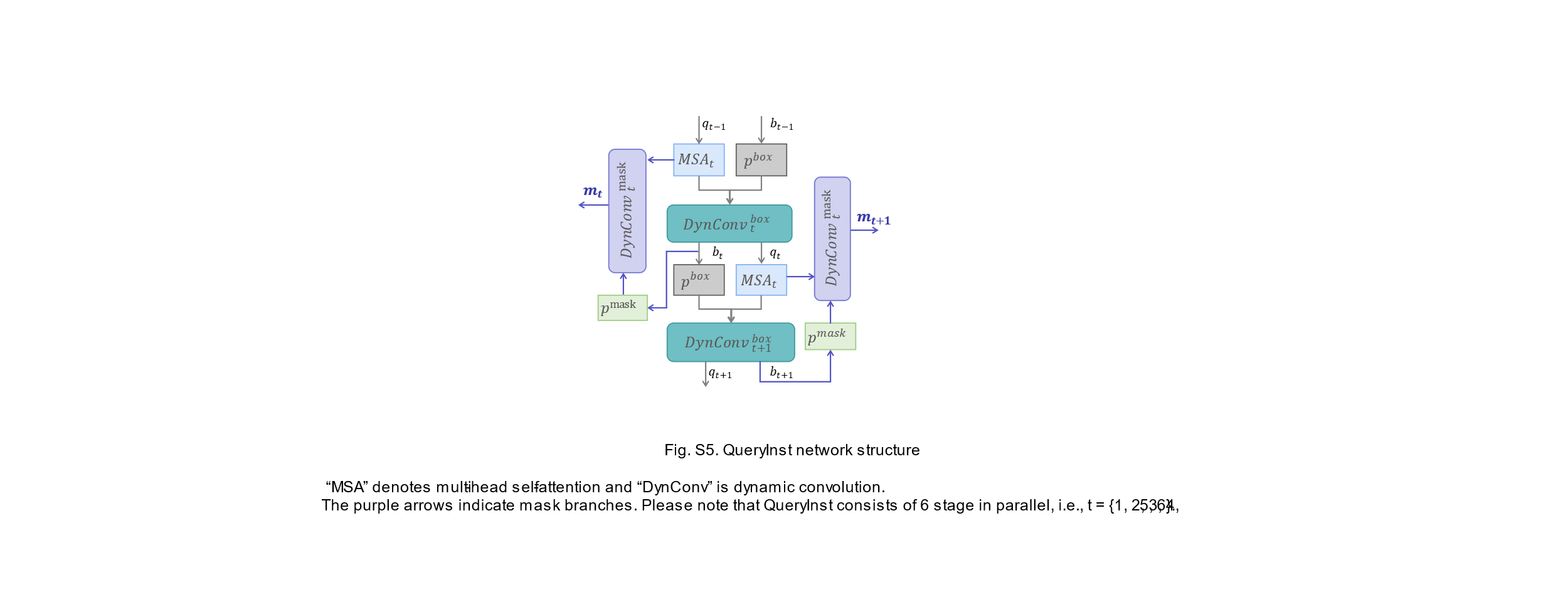


**Fig. S5. QueryInst network structure** “MSA” denotes multi-head self-attention and “DynConv” is dynamic convolution. The purple arrows indicate mask branches. m_t_ is the current stage's mask prediction result and b_t_ is the bounding box predictions from the object detector.. p^mask^ represents a region-wise pooling operator used for extracting mask RoI features and p^box^ utilizes ROI Align operation. Query q is inherited from DEtection Transformer, and it involves initializing N object queries randomly. Using nn.Embedding, N object queries and queries features are initialized. Additionally, the initialized bounding boxes (bbox) are decoded to represent bbox information in the original dimensions. This bbox decoding transforms them from center-point and height-width representation to left-top and right-bottom corner-point representation. Please note that QueryInst consists of 6 stages in parallel, i.e., t = {1, 2, 3, 4, 5, 6}.

**Model 6: ResNet 50+Mask2Former**

Masked-attention Mask Transformer (Mask2Former) [7] is a universal image segmentation model. The low-resolution features are extracted from an image by backbone, a pixel decoder then gradually upsamples low-resolution features from the output of the backbone to generate high-resolution features. The feature pyramid consists of both low- and high-resolution features will then be fed to multiple Transformer decoder as one-resolution-per-decoder basis. Masked attention is used in the Transformer decoder which restricts the attention to localized features centered around predicted segments. Compared to the cross-attention used in a standard Transformer decoder which attends to all locations in an image, the proposed masked attention leads to faster convergence and improved performance.


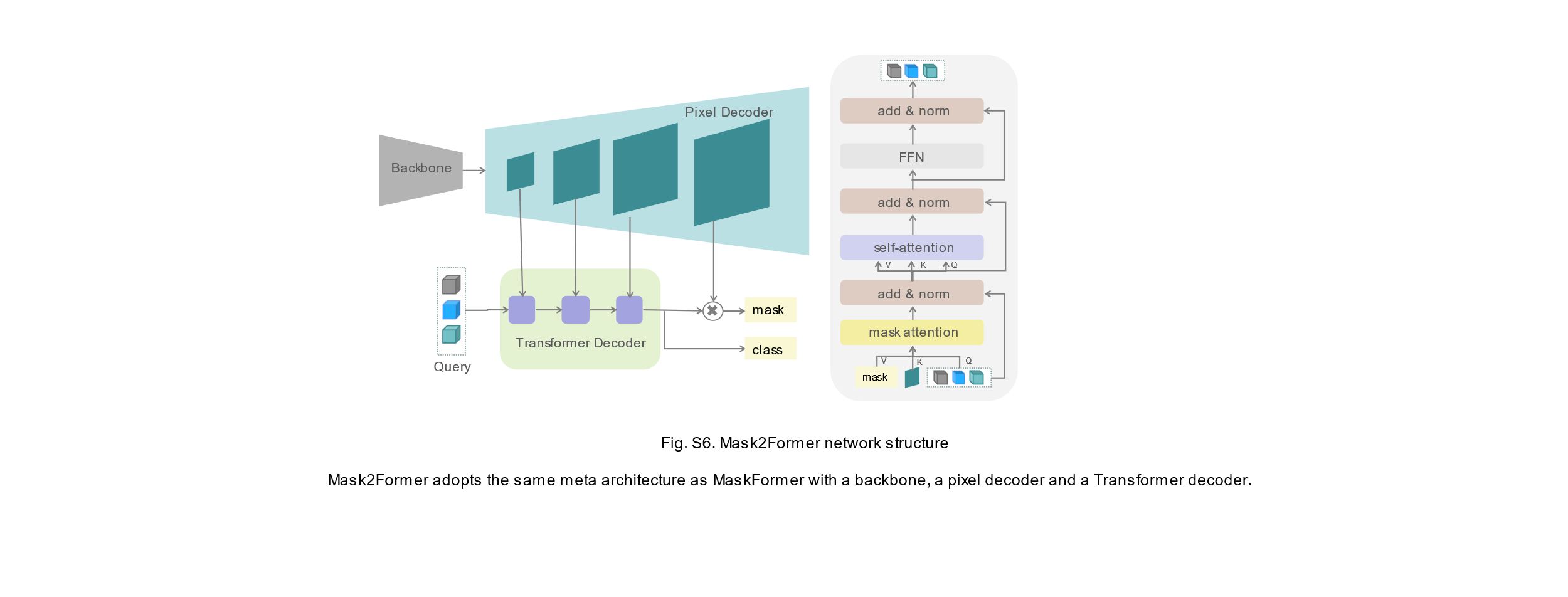


**Fig. S6. Mask2Former network structure** Mask2Former uses the same meta-architecture as MaskFormer with a backbone, a pixel decoder and a Transformer decoder. This Transformer decoder includes a masked attention operator to extract localized features by limiting cross-attention to the predicted mask's foreground region for each query. To handle small objects, an efficient multi-scale strategy is employed, leveraging high-resolution features by feeding feature maps from the pixel decoder's feature pyramid into subsequent Transformer decoder layers. Optimization enhancements are also incorporated to boost model performance without adding extra computational complexity.

**Data Preparation and Augmentation**

To enhance the performance of AI model (i.e. SCNet, shown in **Fig. S4**), we applied data augmentation during training to simulate various image variations (as in **Fig. 2d**). We cropped image patches (648 * 648 pixels at 10x) from the original H&E WSIs and subjected them to random augmentation, which included operations like flipping, orientation changes, Gaussian blurring, brightness/contrast adjustments, and color space transformations (applied to the H channel in HSV space and the R channel in RGB space). This augmentation diversifies the training dataset, reducing the risk of overfitting and bolstering model robustness. More importantly, color augmentation significantly enhances model generalization, enabling its application to both H&E and PAS-stained images effectively. The corresponding annotations were converted into binary image patches for kidney tissue segmentation.

For mesangial and endothelial cells within the glomerular area, we selected and cropped the glomerular region, padding the remaining space to reach a size of 1024*1024 pixels at 40x, ensuring consistent model input dimensions (as shown in **Fig. 2e**). Mesangial and endothelial cells were then extracted from these patches and underwent a similar random augmentation.

Nuclei annotations extracted from “data-science-bowl-2018” underwent similar random augmentation.

**Data Distribution and Experimental Setup**

We extracted 988 Regions of Interest (ROIs) from 779 Whole Slide Images (WSIs) and split them to training, validation, and testing set with a ratio of 7:1:2 for the development and optimization of deep learning model, the detailed distribution of three sets is shown below in **Table S1**. The computational burden of model training is intermediate, as shown in **Table S2**.

**Table S1. Data Distribution**

| Magnification | Patch size | Training | Validation | Testing | Total |
| --- | --- | --- | --- | --- | --- |
| 10X | 648*648*3 | 690 | 98 | 200 | 988 |

**Table S2. Experiment Configuration**

| OS | CPU | Memory | GPU | CUDA | CUDNN | TensorFlow |
| --- | --- | --- | --- | --- | --- | --- |
| Ubuntu16.04 x64 | Intel(R) Xeon(R) CPU E5-2643 v3 @ 3.40GHz | 32GB | GeForce RTX 2080Ti*4 | v10.2 | v7.6.5 | v1.9rc0 |

**Section II: Test results across different models**

**Model Performance**

We reported the receiver operating characteristic (ROC) curve and the area under ROC curve (AUC) of each model as shown in main text **Fig. 4e**. The detailed sensitivity, specificity, and AUC of SCNet for each class are shown here in **Table S3**. Apart from ROC curve, the Precision-Recall (P-R) curve of different models is also reported in **Fig. S7**.

**Table S3. Sensitivity, specificity, and AUC of SCNet for five kidney structures in testing set**

|  | glomerular tuft | glomerular | proximal tubules | distal tubules | arteries |
| --- | --- | --- | --- | --- | --- |
| Sensitivity | 0.998 | 0.994 | 0.926 | 0.915 | 0.948 |
| Specificity | 0.915 | 0.905 | 0.818 | 0.815 | 0.833 |
| AUC | 0.997 | 0.988 | 0.929 | 0.94 | 0.966 |


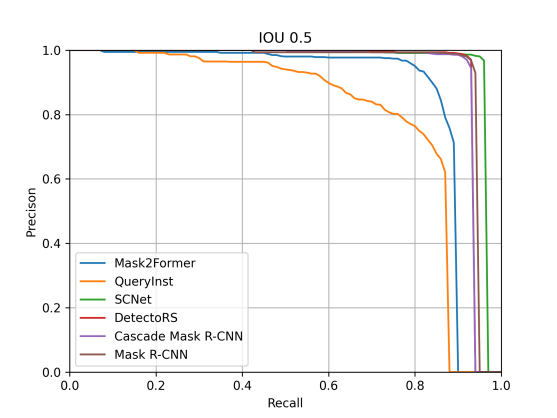


**Fig. S7. P-R curve for six models for optimization.** The kidney structure detection model of the proposed APKD is optimized from six latest CNN models. All six models were based on the ResNet 50 backbone due to its success application in the field. We evaluated the accuracy (in terms of mAP50, which is the mean value of AP50 of each class) of six models in both training set and testing set**.** SCNet with ResNet 50 as the backbone outperformed the other five models and achieved 0.94 mAP50 in testing set.

**Inference Speed and Inference Result**

To assess the speed of inference, we prepared an independent testing set including 200 image patches (648*648 pixel) to input to six models and recorded the inference time. The average inference time for six models is about 0.3 seconds per image on a standard workstation as shown in **Table S4**. Three examples of the inference result of six models are shown in **Fig. S8**, respectively.

**Table S4. Inference time for six models**

| Model | Inference time for testing set (200 images) in second | Input patch size |
| --- | --- | --- |
| Mask R-CNN | 50 | 648*648*3 |
| Cascade Mask R-CNN | 55 | 648*648*3 |
| DetectoRS | 81 | 648*648*3 |
| SCNet | 59 | 648*648*3 |
| QueryInst | 57 | 648*648*3 |
| Mask2Former | 67 | 648*648*3 |


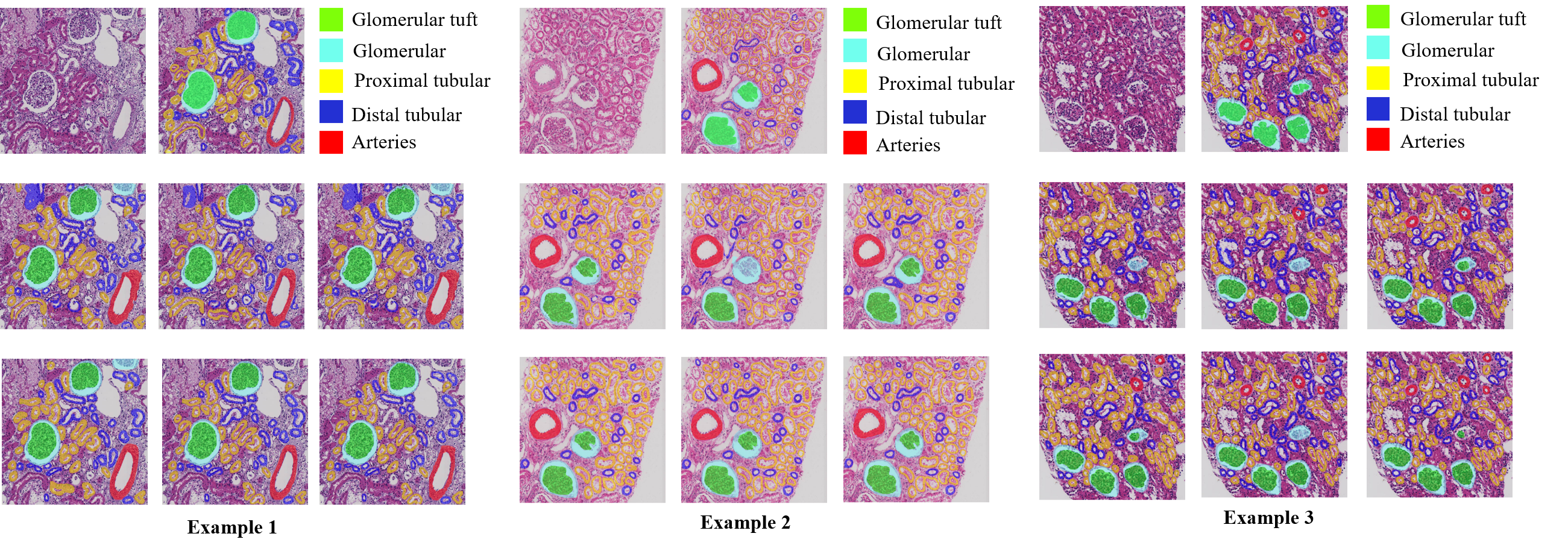
**Fig. S8. Inference result of different models, example 1-3.**

**Correlation and Statistical Analysis**

The age-related studies on the number and size of glomeruli have been reported [8]. However, the quantitative information about the relationship between the number and size of glomeruli and increased age is scant, especially in child cohorts. We analyzed the area of glomeruli detected in a cohort of children aged 1-5, 6-10, 11-15, and 16-20 years using the analysis of variance (ANOVA) testing, as shown in **Table S5**. It revealed a significant increase in glomeruli areas from ages 1-5 until 16-20 years for both females and males, as illustrated in main text **Fig. 6d**.

**Table S5. Analysis of variance (ANOVA) of glomerulus area in a cohort of children**

| *ANOVA - glomerulus mean area* | | | | | | | | | | |
| --- | --- | --- | --- | --- | --- | --- | --- | --- | --- | --- |
| Cases | | Sum of Squares | | df | Mean Square | | | F | | p |
| Age Range | | 5.872e +9 | | 3 | 1.9575e +9 | | | 133.683 | | < .001 |
| Gender | | 4.185e +7 | | 1 | 4.185e +7 | | | 2.858 | | 0.091 |
| Age Range ✻ Gender | | 1.668e +7 | | 3 | 5.560e +6 | | | 0.380 | | 0.768 |
| Residuals | | 3.050e +10 | | 2083 | 1.4646e +7 | | |  | |  |
| *Post Hoc Comparisons - Age Range* | | | | | | | | | | |
|  |  | | Mean Difference | | | SE | t | | p_tukey_ | |
| 1_to_5 | 11_to_15 | | -4151.64 | | | 277.864 | -14.941 | | < .001*** | |
|  | 16_to_20 | | -6588.37 | | | 412.279 | -15.98 | | < .001*** | |
|  | 6_to_10 | | -1938.19 | | | 272.427 | -7.115 | | < .001*** | |
| 11_to_15 | 16_to_20 | | -2436.73 | | | 363.568 | -6.702 | | < .001*** | |
|  | 6_to_10 | | 2213.452 | | | 190.851 | 11.598 | | < .001*** | |
| 16_to_20 | 6_to_10 | | 4650.183 | | | 359.43 | 12.938 | | < .001*** | |
| *** p < .001 | | | | | | | | | | |
| Note. P-value adjusted for comparing a family of 4 | | | | | | | | | | |
| Note. Results are averaged over the levels of: Gender | | | | | | | | | | |

We have also analyzed the total number of cells in glomerular tuft in various kidney diseases using ANOVA test as shown in **Table S6**. We observed that the number of cells in the glomerular tuft of endocapillary proliferative glomerulonephritis (EPGN) is significantly higher than that of other diseases as illustrated in main text **Fig. 6e**.

**Table S6. Analysis of variance (ANOVA) of number of cells in the glomerular tuft in paediatric kidney disease**

| *ANOVA - Average number of cells in glomerular tuft* | | | | | | | | | | |
| --- | --- | --- | --- | --- | --- | --- | --- | --- | --- | --- |
| Cases | | | Sum of Squares | df | | Mean Square | | F | | p |
| Paediatric kidney disease | | | 52168.21 | 9 | | 5796.468 | | 5.723 | | < .001 |
| Gender | | | 1505.864 | 1 | | 1505.864 | | 1.487 | | 0.223 |
| Paediatric kidney disease ✻ Gender | | | 11435.54 | 9 | | 1270.615 | | 1.254 | | 0.257 |
| Residuals | | | 2.69E+06 | 2659 | | 1012.884 | |  | |  |
| *Post Hoc Comparisons - paediatric kidney disease* | | | | | | | | | | |
|  |  | Mean Difference | | | SE | | t | | p_tukey_ | |
| EPGN | CG | -23.353 | | | 6.408 | | -3.644 | | 0.01* | |
| EPGN | FSGS | 31.038 | | | 8.582 | | 3.617 | | 0.011* | |
| EPGN | HSPN | 24.063 | | | 4.146 | | 5.804 | | < .001*** | |
| EPGN | IgAN | 24.657 | | | 4.374 | | 5.638 | | < .001*** | |
| EPGN | MCG | 27.198 | | | 4.129 | | 6.586 | | < .001*** | |
| EPGN | MN | 30.314 | | | 7.266 | | 4.172 | | 0.001** | |
| EPGN | MPGN | 19.462 | | | 4.518 | | 4.308 | | < .001*** | |
| EPGN | MsPGN | 23.969 | | | 4.233 | | 5.663 | | < .001*** | |
| ** p < .01, *** p < .001 | | | | | | | | | | |
| Note. P-value adjusted for comparing a family of 10 | | | | | | | | | | |
| Note. Results are averaged over the levels of: Gender | | | | | | | | | | |

**Section III: Common pathological features extracted using APKD**

We extracted 58 comprehensive pathomorphological features with the help of APKD model to aid early diagnosis and prognosis, the definition of features is listed below in **Table S7**.

**Table S7. Pathomorphological features extracted by APKD and their description**

| No. | Features | Detail |
| --- | --- | --- |
| 1 | glom_mean_cell_num | Average cell mass of glomerular |
| 2 | glom_max_cell_num | Glomerular maximum cell mass |
| 3 | glom_mean_area | Average glomerular area |
| 4 | glom_max_area | Maximum glomerular area |
| 5 | glom_mean_cir | Mean glomerular circumference |
| 6 | glom_max_cir | Maximum circumference of glomerular |
| 7 | glom_mean_round_rate | Mean roundness of glomerular |
| 8 | glom_max_round_rate | Maximum circularity of glomerular |
| 9 | tuft_mean_area | Mean glomerular tuft area |
| 10 | tuft_max_area | Largest glomerular tuft area |
| 11 | tuft_mean_cir | Mean Glomerular tuft Circumference |
| 12 | tuft_max_cir | Glomerular tuft maximum circumference |
| 13 | tuft_mean_round_rate | Mean roundness of glomerular tuft |
| 14 | tuft_max_round_rate | Glomerular tuft maximum roundness |
| 15 | glom_mean_round_baoshi_core_num | Mean cell mass in Bowman's capsule |
| 16 | glom_max_round_baoshi_core_num | Maximum cell mass in the glomerular Bowman's capsule |
| 17 | glom_mean_empty_rate | Mean cavity ratio in glomeruli |
| 18 | glom_max_empty_rate | The proportion of the largest cavity in the glomerulus |
| 19 | glom_mean_max_radius | Mean glomerular diameter |
| 20 | glom_max_max_radius | Maximum glomerular diameter |
| 21 | glom_mean_xinyue | Number of crescents/number of glomeruli in WSI |
| 22 | glom_max_xinyue | Number of crescents in WSI |
| 23 | nt_mean_area | Mean area of ​​proximal convoluted tubules |
| 24 | nt_max_area | Maximum area of ​​proximal convoluted tubules |
| 25 | nt_mean_out_area | Mean outer contour area of ​​proximal convoluted tubules |
| 26 | nt_max_out_area | Maximum outer contour area of ​​proximal convoluted tubules |
| 27 | nt_mean_in_area | Mean inner contour area of ​​proximal convoluted tubules |
| 28 | nt_max_in_area | Maximum inner contour area of ​​proximal convoluted tubules |
| 29 | nt_mean_out_length | Mean outer contour circumference of proximal convoluted tubules |
| 30 | nt_max_out_length | Maximum outer contour circumference of proximal convoluted tubule |
| 31 | nt_mean_in_length | Mean inner contour circumference of proximal convoluted tubules |
| 32 | nt_max_in_length | Maximum inner contour circumference of proximal convoluted tubule |
| 33 | nt_mean_round_rate | Average roundness of distal convoluted tubules |
| 34 | nt_max_round_rate | The maximum roundness of the distal convoluted tubule |
| 35 | ft_mean_area | Mean area of ​​distal convoluted tubules |
| 36 | ft_max_area | Maximum area of ​​distal convoluted tubule |
| 37 | ft_mean_out_area | Mean outer contour area of ​​distal convoluted tubules |
| 38 | ft_max_out_area | Maximum outer contour area of ​​distal convoluted tubule |
| 39 | ft_mean_in_area | Mean inner contour area of ​​distal convoluted tubules |
| 40 | ft_max_in_area | Maximum inner contour area of ​​distal convoluted tubules |
| 41 | ft_mean_out_length | Mean outer contour circumference of distal convoluted tubules |
| 42 | ft_max_out_length | Maximum outer contour circumference of distal convoluted tubule |
| 43 | ft_mean_in_length | Mean inner contour circumference of distal convoluted tubules |
| 44 | ft_max_in_length | Maximum inner contour circumference of distal convoluted tubule |
| 45 | ft_mean_round_rate | Average roundness of distal convoluted tubules |
| 46 | ft_max_round_rate | The maximum roundness of the distal convoluted tubule |
| 47 | cert_mean_area | Mean area of ​​​​arteries |
| 48 | cert_max_area | Maximum area of ​​arteries |
| 49 | cert_mean_out_area | Mean outer contour area of ​​arteries |
| 50 | cert_max_out_area | Maximum outer contour area of ​​​​arteries |
| 51 | cert_mean_in_area | Mean inner contour area of ​​arteries |
| 52 | cert_max_in_area | Maximum inner contour area of ​​arteries |
| 53 | cert_mean_out_length | Mean outer contour circumference of arteries |
| 54 | cert_max_out_length | Maximum outer contour circumference of arteries |
| 55 | cert_mean_in_length | Mean inner contour circumference of arteries |
| 56 | cert_max_in_length | Maximum inner contour circumference of arteries |
| 57 | cert_mean_round_rate | Mean roundness of arteries |
| 58 | cert_max_round_rate | Maximum circularity of arteries |

**Section IV: Summary of recent publication on deep learning based kidney pathological diagnosis**

Recently, an accumulation of kidney pathological diagnosis has been reported based on deep learning, **Table S8** shows a summary of the applications of deep learning in nephropathology.

**Table S8: Current applications of Deep Learning in nephropathology**

| Reference | Methods | Imaging | Species | Tasks |
| --- | --- | --- | --- | --- |
| [9] | HOG+SVM | IHC | rat | Glomerulus Detection |
| [10] | Icy & Cytomine | TRI | human | Glomerulus Detection |
| [11] | Gabor texture segmentation & bottleneck detection | HE, PAS | rat | Glomerulus segmentation |
| [12] | CNN | Jones HE, PAS,  Sirius red, CD10 | human | Glomerulus Detection |
| [13] | U-Net | PAS | mouse | Glomerulus Detection,segmentation |
| [14] | PathoSpotter-K (kNN) | PAS, HE | human | Classification proliferative glomerular lesions |
| [15] | CNN | Frozen HE | human | Glomerulus segmentation, classification (normal, sclerosis) |
| [16] | CNN | PAS | human | Glomerulus Detection, Classification |
| [17] | Faster RCNN | TRI | rat/human | Classification glomerulus/non-glomerulus |
| [18] | CNN | TRI | human | Classsification Interstitial fibrosis |
| [19] | LBP+SVM | HE, PAS, SIL, TRI | mouse, rat,  human | Glomerulus detection |
| [20]&[21] | DNNs | PAS | mouse | Glomerulus segmentation, quantified mesangial matrix proliferation, numbers of nuclei, and capillary openness. |
| [22] | Inception V3 CNN | TRI | human | Glomerulus detection, segmentation (globally sclerosed) |
| [23] | CNN | PAS | human | Segmentation (glomeruli, tubuli, and interstitium) |
| [24] | CNN | PAS | human | Quantify glomeruli: nuclei, capillary lumina and Bowman spaces |
| [25] | CNN/SegNet | PAS | human | Classification, segmentation glomeruli normal/sclerosed |
| [26] | CNN | PAS | human | Identification, classification (glomerulus, intrinsic glomerular cell recognition) |
| [27] | InceptionV3-CNN | PAS, SIL | human | Classification seven glomerular pathological findings |
| [28] | CNN | IF | human | IgG, IgA, IgM, C1q and C3 complement fractions, fibrinogen, and κ- and λ-light chains |
| [29] | CNN | HE, PAS | human | Classification Hypercellularity (mesangial, endocapilar) |
| [30] | VGG16 | Frozen HE | human | quantify glomerulosclerosis |
| [31] | U-Net | HE, PAS, SIL, TRI | human | Detection, Segmentation quantification (Glomeruli, tubular, capillaries, arteries) |
| [32] | CNN | PAS | bears, pigs,  marmosets, mouse, rats | Segmentation (Different experimental disease models) |
| [33] | U-Net | PAS, HE | human | Segmentation,classification(normal,sclerosis) quantify glomerulosclerosis |
| [34] | CNN | PAS | human | Detection, segmentation (interstitial fibrosis, tubular atrophy, glomerulosclerosis) |
| [35] | CNN | Masson | human | Glomerular volume, glomerular density, interstitial fibrosis, tubular atrophy, vascular intimal thickness |
| [36] | R-CNN | PAS | human | Quantify interstitium, tubules, mononuclear leukocyte infiltration |

**CNN**: convolutional neural network; **HOG**: histogram of oriented gradients; **SVM**: support vector machine; **LBP**: local binary patterns; **FCNN**: fully convolutional network; **IHC**: immunohistochemistry; **HE**: hematoxylin and eosin; **TRI**: trichrome Masson; **PAS**: periodic acid-Schiff; **SIL**: periodic acid-methenamine silver; **IF**: immunofluorescence.

**Reference**

1. He, K., Zhang, X., Ren, S., & Sun, J. (2016). Deep residual learning for image recognition. In Proceedings of the IEEE conference on computer vision and pattern recognition (pp. 770-778).
2. He, K., Gkioxari, G., Dollár, P., & Girshick, R. (2017). Mask r-cnn. In Proceedings of the IEEE international conference on computer vision (pp. 2961-2969).
3. Cai, Z., & Vasconcelos, N. (2018). Cascade r-cnn: Delving into high quality object detection. In Proceedings of the IEEE conference on computer vision and pattern recognition (pp. 6154-6162).
4. Qiao, S., Chen, L. C., & Yuille, A. (2021). Detectors: Detecting objects with recursive feature pyramid and switchable atrous convolution. In Proceedings of the IEEE/CVF conference on computer vision and pattern recognition (pp. 10213-10224).
5. Vu, T., Kang, H., & Yoo, C. D. (2021, May). Scnet: Training inference sample consistency for instance segmentation. In Proceedings of the AAAI Conference on Artificial Intelligence (Vol. 35, No. 3, pp. 2701-2709).
6. Fang, Y., Yang, S., Wang, X., Li, Y., Fang, C., Shan, Y., ... & Liu, W. (2021). Instances as queries. In Proceedings of the IEEE/CVF International Conference on Computer Vision (pp. 6910-6919).
7. Cheng, B., Misra, I., Schwing, A. G., Kirillov, A., & Girdhar, R. (2022). Masked-attention mask transformer for universal image segmentation. In Proceedings of the IEEE/CVF Conference on Computer Vision and Pattern Recognition (pp. 1290-1299).
8. Nyengaard, J. R., & Bendtsen, T. F. (1992). Glomerular number and size in relation to age, kidney weight, and body surface in normal man. The Anatomical Record, 232(2), 194-201.
9. Kato, T., Relator, R., Ngouv, H., Hirohashi, Y., Takaki, O., Kakimoto, T., & Okada, K. (2015). Segmental HOG: new descriptor for glomerulus detection in kidney microscopy image. *Bmc Bioinformatics*, *16*(1), 1-16.
10. Marée, R., Dallongeville, S., Olivo-Marin, J. C., & Meas-Yedid, V. (2016, April). An approach for detection of glomeruli in multisite digital pathology. In *2016 IEEE 13th International Symposium on Biomedical Imaging (ISBI)* (pp. 1033-1036). IEEE.
11. Sarder, P., Ginley, B., & Tomaszewski, J. E. (2016, March). Automated renal histopathology: digital extraction and quantification of renal pathology. In *Medical Imaging 2016: Digital Pathology* (Vol. 9791, pp. 112-123). SPIE.
12. Temerinac-Ott, M., Forestier, G., Schmitz, J., Hermsen, M., Bräsen, J. H., Feuerhake, F., & Wemmert, C. (2017, September). Detection of glomeruli in renal pathology by mutual comparison of multiple staining modalities. In *Proceedings of the 10th International Symposium on Image and Signal Processing and Analysis* (pp. 19-24). IEEE.
13. Gadermayr, M., Gupta, L., Appel, V., Boor, P., Klinkhammer, B. M., & Merhof, D. (2019). Generative adversarial networks for facilitating stain-independent supervised and unsupervised segmentation: a study on kidney histology. *IEEE transactions on medical imaging*, *38*(10), 2293-2302.
14. Barros, G. O., Navarro, B., Duarte, A., & Dos-Santos, W. L. (2017). PathoSpotter-K: A computational tool for the automatic identification of glomerular lesions in histological images of kidneys. *Scientific reports*, *7*(1), 1-8.
15. Marsh, J. N., Matlock, M. K., Kudose, S., Liu, T. C., Stappenbeck, T. S., Gaut, J. P., & Swamidass, S. J. (2018). Deep learning global glomerulosclerosis in transplant kidney frozen sections. *IEEE transactions on medical imaging*, *37*(12), 2718-2728.
16. Gallego, J., Pedraza, A., Lopez, S., Steiner, G., Gonzalez, L., Laurinavicius, A., & Bueno, G. (2018). Glomerulus classification and detection based on convolutional neural networks. *Journal of Imaging*, *4*(1), 20.
17. Bukowy, J. D., Dayton, A., Cloutier, D., Manis, A. D., Staruschenko, A., Lombard, J. H., ... & Cowley, A. W. (2018). Region-based convolutional neural nets for localization of glomeruli in trichrome-stained whole kidney sections. *Journal of the American Society of Nephrology*, *29*(8), 2081-2088.
18. Kolachalama, V. B., Singh, P., Lin, C. Q., Mun, D., Belghasem, M. E., Henderson, J. M., ... & Chitalia, V. C. (2018). Association of pathological fibrosis with renal survival using deep neural networks. *Kidney international reports*, *3*(2), 464-475.
19. Simon, O., Yacoub, R., Jain, S., Tomaszewski, J. E., & Sarder, P. (2018). Multi-radial LBP features as a tool for rapid glomerular detection and assessment in whole slide histopathology images. *Scientific reports*, *8*(1), 1-11.
20. Sheehan, S. M., & Korstanje, R. (2018). Automatic glomerular identification and quantification of histological phenotypes using image analysis and machine learning. *American Journal of Physiology-Renal Physiology*, *315*(6), F1644-F1651.
21. Sheehan, S., Mawe, S., Cianciolo, R. E., Korstanje, R., & Mahoney, J. M. (2019). Detection and classification of novel renal histologic phenotypes using deep neural networks. *The American Journal of Pathology*, *189*(9), 1786-1796.
22. Kannan, S., Morgan, L. A., Liang, B., Cheung, M. G., Lin, C. Q., Mun, D., ... & Kolachalama, V. B. (2019). Segmentation of glomeruli within trichrome images using deep learning. *Kidney international reports*, *4*(7), 955-962.
23. Hermsen, M., de Bel, T., Den Boer, M., Steenbergen, E. J., Kers, J., Florquin, S., ... & van der Laak, J. A. (2019). Deep learning–based histopathologic assessment of kidney tissue. *Journal of the American Society of Nephrology*, *30*(10), 1968-1979.
24. Ginley, B., Lutnick, B., Jen, K. Y., Fogo, A. B., Jain, S., Rosenberg, A., ... & Sarder, P. (2019). Computational segmentation and classification of diabetic glomerulosclerosis. *Journal of the American Society of Nephrology*, *30*(10), 1953-1967.
25. Bueno, G., Gonzalez-Lopez, L., Garcia-Rojo, M., Laurinavicius, A., & Deniz, O. (2020). Data for glomeruli characterization in histopathological images. *Data in brief*, *29*, 105314.
26. Zeng, C., Nan, Y., Xu, F., Lei, Q., Li, F., Chen, T., ... & Liu, Z. (2020). Identification of glomerular lesions and intrinsic glomerular cell types in kidney diseases via deep learning. *The Journal of pathology*, *252*(1), 53-64.
27. Uchino, E., Suzuki, K., Sato, N., Kojima, R., Tamada, Y., Hiragi, S., ... & Okuno, Y. (2020). Classification of glomerular pathological findings using deep learning and nephrologist–AI collective intelligence approach. *International Journal of Medical Informatics*, *141*, 104231.
28. Ligabue, G., Pollastri, F., Fontana, F., Leonelli, M., Furci, L., Giovanella, S., ... & Magistroni, R. (2020). Evaluation of the classification accuracy of the kidney biopsy direct immunofluorescence through convolutional neural networks. *Clinical Journal of the American Society of Nephrology*, *15*(10), 1445-1454.
29. Chagas, P., Souza, L., Araújo, I., Aldeman, N., Duarte, A., Angelo, M., ... & Oliveira, L. (2020). Classification of glomerular hypercellularity using convolutional features and support vector machine. *Artificial intelligence in medicine*, *103*, 101808.
30. Marsh, J. N., Liu, T. C., Wilson, P. C., Swamidass, S. J., & Gaut, J. P. (2021). Development and validation of a deep learning model to quantify glomerulosclerosis in kidney biopsy specimens. *JAMA network open*, *4*(1), e2030939-e2030939.
31. Jayapandian, C. P., Chen, Y., Janowczyk, A. R., Palmer, M. B., Cassol, C. A., Sekulic, M., ... & Lin, J. J. (2021). Development and evaluation of deep learning–based segmentation of histologic structures in the kidney cortex with multiple histologic stains. *Kidney international*, *99*(1), 86-101.
32. Bouteldja, N., Klinkhammer, B. M., Bülow, R. D., Droste, P., Otten, S. W., von Stillfried, S. F., ... & Merhof, D. (2021). Deep learning–based segmentation and quantification in experimental kidney histopathology. *Journal of the American Society of Nephrology*, *32*(1), 52-68.
33. Gallego, J., Swiderska-Chadaj, Z., Markiewicz, T., Yamashita, M., Gabaldon, M. A., & Gertych, A. (2021). A U-Net based framework to quantify glomerulosclerosis in digitized PAS and H&E stained human tissues. *Computerized Medical Imaging and Graphics*, *89*, 101865.
34. Ginley, B., Jen, K. Y., Han, S. S., Rodrigues, L., Jain, S., Fogo, A. B., ... & Sarder, P. (2021). Automated computational detection of interstitial fibrosis, tubular atrophy, and glomerulosclerosis. *Journal of the American Society of Nephrology*, *32*(4), 837-850.
35. Marechal, E., Jaugey, A., Tarris, G., Paindavoine, M., Seibel, J., Martin, L., ... & Legendre, M. (2022). Automatic Evaluation of Histological Prognostic Factors Using Two Consecutive Convolutional Neural Networks on Kidney Samples. *Clinical Journal of the American Society of Nephrology*, *17*(2), 260-270.
36. Yi, Z., Salem, F., Menon, M. C., Keung, K., Xi, C., Hultin, S., ... & Zhang, W. (2022). Deep learning identified pathological abnormalities predictive of graft loss in kidney transplant biopsies. *Kidney International*, *101*(2), 288-298.
